# Effects of Prenatal Exposure to Inflammation Coupled With Stress Exposure During Adolescence on Cognition and Synaptic Protein Levels in Aged CD-1 Mice

**DOI:** 10.3389/fnagi.2020.00157

**Published:** 2020-07-06

**Authors:** Zhe-Zhe Zhang, Zhan-Qiang Zhuang, Shi-Yu Sun, He-Hua Ge, Yong-Fang Wu, Lei Cao, Lan Xia, Qi-Gang Yang, Fang Wang, Gui-Hai Chen

**Affiliations:** ^1^Department of Neurology or Department of Critical Care, The First Affiliated Hospital of Anhui Medical University, Hefei, China; ^2^Department of Neurology (Sleep Disorders), The Affiliated Chaohu Hospital of Anhui Medical University, Hefei, China; ^3^Department of Neurology, The Second Affiliated Hospital of Anhui Medical University, Hefei, China

**Keywords:** aging, learning and memory, stress, synaptic proteins, mice

## Abstract

Age-associated impairment of spatial learning and memory (AISLM) presents substantial challenges to our health and society. Increasing evidence has indicated that embryonic exposure to inflammation accelerates the AISLM, and this can be attributable, at least partly, to changed synaptic plasticity associated with the activities of various proteins. However, it is still uncertain whether social psychological factors affect this AISLM and/or the expression of synaptic protein-associated genes. Synaptotagmin-1 (Syt1) and activity-regulated cytoskeleton-associated protein (Arc) are two synaptic proteins closely related to cognitive functions. In this study, pregnant CD-1 mice received daily intraperitoneal injections of lipopolysaccharide (LPS) (50 μg/kg) or normal saline at days 15–17 of gestation, and half of the offspring of each group were then subjected to stress for 28 days in adolescence. The Morris water maze (MWM) test was used to separately evaluate spatial learning and memory at 3 and 15 months of age, while western blotting and RNAscope assays were used to measure the protein and mRNA levels of Arc and Syt1 in the hippocampus. The results showed that, at 15 months of age, control mice had worse cognitive ability and higher protein and mRNA levels of Arc and Syt1 than their younger counterparts. Embryonic exposure to inflammation or exposure to stress in adolescence aggravated the AISLM, as well as the age-related increase in Arc and Syt1 expression. Moreover, the hippocampal protein and mRNA levels of Arc and Syt1 were significantly correlated with the performance in the learning and memory periods of the MWM test, especially in the mice that had suffered adverse insults in early life. Our findings indicated that prenatal exposure to inflammation or stress exposure in adolescence exacerbated the AISLM and age-related upregulation of Arc and Syt1 expression, and these effects were linked to cognitive impairments in CD-1 mice exposed to adverse factors in early life.

## Introduction

Age-associated impairment of spatial learning and memory (AISLM) is a common phenomenon in humans and presents a substantial challenge to our health and society (Foster, 2012). However, the factors and neurobiological mechanisms underlying AISLM remain poorly understood. In humans, viral or bacterial infections during pregnancy can delay the normal development of the central nervous system in the offspring (Watanabe et al., 2010; Diz-Chaves et al., 2013). Chronic inflammation due to maternal infection during pregnancy contributes to the activation of astroglia and microglia and subsequently an increase in the production of proinflammatory cytokines in the brains of offspring. Studies using animal models have shown that inhibition of astrocytic activation through administration of minocycline, an anti-inflammatory, contributes to relieving postoperative cognitive impairment in aged mice (Jin et al., 2014). Lipopolysaccharide (LPS), the main toxic components of the cell wall of Gram-negative bacteria and other microorganisms such as *Chlamydia*, *Rickettsia*, and the spirochetes, can induce neuroinflammatory responses, including activation and proliferation of microglia and excessive production of proinflammatory cytokines such as interleukin-6, interferon-gamma, and tumor necrosis factor (Dantzer, 2001). These cytokines can affect the normal function of the brain and accelerate brain aging through specific signaling pathways (Schlotz and Phillips, 2009; Akbarian et al., 2013; Patterson, 2015). These proinflammatory factors can also enter the fetal blood circulation and brain through the placental barrier and the blood–brain barrier, leading to increased levels of inflammatory cytokines in the fetal brain. Increased inflammatory cytokine levels can stimulate glial cells, thereby causing lasting adverse effects on fetal growth, fetal development, and postnatal neurobehaviors, or lead to accelerated aging of progeny and AISLM (Guzowski et al., 2001; Arrode-Brusés and Brusés, 2012; Chang et al., 2018). This may align with an age-related decline in synaptic function, such as damaged synaptic plasticity and neurotransmission, in some brain regions (McAfoose and Baune, 2009; Nolan et al., 2011). These changes can impair the synaptic connections between axonal buttons and dendritic spines by affecting the levels of synaptic proteins or the size and number of synapses or dendritic spines in neurons (Zhu et al., 2015). We have previously shown that exposure to LPS during pregnancy can accelerate AISLM in middle-aged CD-1 mice (Li X. Y. et al., 2016) and that maternal inflammation due to LPS administration during pregnancy can affect AISLM in their offspring from midlife to senectitude (Li X. W. et al., 2016). Moreover, the levels of some synaptic proteins in the hippocampus also change with age, such as an increase in the levels of synaptotagmin-1 (Syt1) and a decrease in those of syntaxin-1 (Li X. W. et al., 2016). Together, these observations indicate that early life exposure to inflammation has a long-term effect on AISLM, especially after midlife.

Stress is a commonly encountered psychosocial factor. Maternal stress during pregnancy can affect fetal brain development and exert profound neurobiological effects on postnatal motor and affective development (Wadhwa et al., 2001; Huizink et al., 2003). Studies have shown that dams suffering stress during pregnancy exhibit hippocampal neuronal loss and reduced neurogenesis (Zhu et al., 2004; Lucassen et al., 2009), while prenatal stress in the rat causes long-term spatial memory deficits and hippocampal abnormalities as detected by magnetic resonance imaging (Liu et al., 2011). These studies on animals demonstrate the importance of exploring whether stress in adolescence accelerates the AISLM induced by prenatal exposure to inflammation.

Synaptic function is extensively impaired with aging, especially in the hippocampus, and may contribute to abnormal neuronal activity and ultimately to cognitive impairment (Vanguilder and Freeman, 2011; Bettio et al., 2017). This synaptic dysfunction may be associated, at least partially, with altered synaptic plasticity resulting from changed hippocampal levels of synaptic proteins such as Syt1, Munc18-1, and SNAP-25, which are associated with AISLM (Cao et al., 2012; Deak and Sonntag, 2012; Zhao et al., 2017). Although numerous synaptic proteins have been identified, which of these may be involved in AISLM remains poorly understood.

Synaptotagmin-1 is abundantly localized at presynaptic vesicles in the brain and acts as the major calcium sensor for mediating fast and synchronous neurotransmitter release (Inoue et al., 2015; Chang et al., 2018). This indicates that Syt1 is important for cognitive function, and further suggests that it may also play a role in AISLM. Additionally, Syt1 expression in the brain can be adversely affected by factors such as aging, inflammation, and stress. Current evidence suggests that dorsal hippocampal levels of Syt1 increase with age, as demonstrated in SAMP8 and CD-1 mice, and this increase is correlated with AISLM (Chen et al., 2007; Li X. W. et al., 2016). Moreover, maternal exposure to LPS during pregnancy can exacerbate age-related upregulation of hippocampal levels of Syt1 in both the mother and offspring (Li X. W. et al., 2016; Li X. Y. et al., 2016). However, it is not known whether prenatal exposure to inflammation affects *Syt1* gene transcription, or how stress during adolescence affects the normal or accelerated age-related increase in Syt1 protein expression.

Activity-regulated cytoskeleton-associated protein (Arc, also known as Arg3.1) is a postsynaptic protein that shuttles between dendrites and nuclear compartments, and is essential for synaptic plasticity and synapse elimination (Barylko et al., 2018; Epstein and Finkbeiner, 2018; Newpher et al., 2018). Arc controls the transport of AMPA receptors (AMPARs), thereby regulating synaptic plasticity, and is necessary for spatial memory consolidation (Waung et al., 2008; Jakkamsetti et al., 2013). However, the relationship between the changes of Arc and cognition is difficult to draw. For instance, increased Arc levels may interfere with learning by altering the shape of spinous processes in transgenic mice (Kelly and Deadwyler, 2003). However, reducing Arc levels using genetics or antisense oligonucleotides also leads to impaired long-term memory formation (Plath et al., 2006; Ploski et al., 2008). In addition, a few studies have indicated that Arc expression in the brain can be affected by factors such as early life experience, age, and cranial irradiation. In Wistar rats, hippocampal Arc expression declines with age, and stress due to separation from the mother in the neonatal period exacerbates this age-related reduction (Solas et al., 2010). To date, it is not known whether prenatal exposure to inflammation changes Arc expression in the brain at different ages, and how stress in adolescence affects normal age-related Arc expression.

Accordingly, in this study, we explored whether prenatal exposure to inflammation either with or without stress during adolescence affected spatial learning and memory in young (3 months old) or aged (15 months old) CD-1 mice. We also evaluated whether the protein and mRNA levels of Syt1 and Arc were altered in different hippocampal subregions of mice of different ages and treatments. Finally, we determined the correlations between spatial learning and memory and the measured neurobiological indicators in the groups of different ages and treatments.

## Materials and Methods

### Animals and Drugs

**FIGURE 1:**
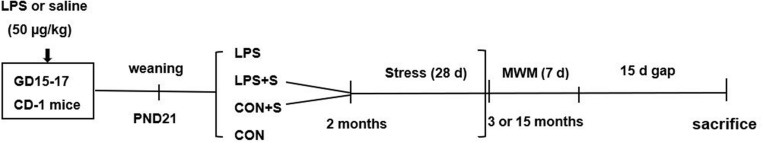
Timeline of experimental events. Pregnant mice were intraperitoneally injected with LPS or normal saline on days 15–17 of gestation (GD). All male offspring were weaned at postnatal day (PND) 21, and were divided into four groups. Between 2 and 3 months of age, the CON + S and LPS + S groups underwent variable stress treatment for 28 days. The MWM test was performed at 3 months (3 M) and 15 months (15 M) PND. Fifteen days after the MWM test, the mice were sacrificed for subsequent biochemical experiments. CON, untreated control group; LPS, lipopolysaccharide treatment group; S, group of mice exposed to stress; MWM, Morris water maze.

All CD-1 mice (6 weeks old, 20 males and 40 females) were purchased from the Medical Experimental Animal Center of Anhui Province, China. Adaptive feeding was provided for 2 weeks before the experiment. The mice were maintained under a 12-h light/dark schedule (lights on at 07:00) and a temperature of 24 ± 1°C with 55 ± 5% humidity. Males and females were mated at a 1:2 ratio. The next day, the presence of a vaginal plug was designated as gestational day (GD) 0. During GDs 15–17, the mice received a daily intraperitoneal injection of LPS (50 μg/kg) or the same volume of normal saline. After normal childbirth and breastfeeding, offspring were separated from their mothers on postnatal day 21. The offspring of mothers that had received LPS were randomly assigned into two groups (LPS groups), one of which was additionally exposed to stress for 28 days from 2 months of age (LPS + S group). The offspring of mothers receiving normal saline were randomly assigned into two control (CON) groups, one of which was additionally exposed to stress in adolescence (CON + S) for 28 days from 2 months of age. When they reached 3 and 15 months, six males from each group were used for experiments. The schematic representation of the experimental timeline was shown in Figure 1. All animal procedures were performed in compliance with the guidelines published in the National Institutes of Health (NIH) Guide for the Care and Use of Laboratory Animals. The protocol was approved by the Experimental Animal Ethics Committee of Anhui Medical University (No. LLSC20160338).

### Stress Induction

The stress scheme was performed according to our previous study (Li et al., 2018). The LPS + S and CON + S groups were randomly and daily exposed to one of the following stress modes. They comprised of binding, suspension, lighting, or fasting at night. Four days constituted one cycle, and it lasted for seven cycles. For binding, the mice were fixed in a mesh made of soft wire (35 × 40 cm) to restrict their movement, resulting in psychological anxiety that did not affect their breathing. Binding lasted 30 min on the first day, and was increased by 10 min each time in the next time of binding. In the suspension test, the tail of the mouse was fixed on a crossbar (1.2 m in height) for 30 min on the first day and then increased by 10 min each time in the next time of suspension. For lighting at night, the light was switched on for 30 min at 30-min intervals from 19:00 to 07:00 the next morning. During fasting at night, the feed was removed from 19:00 to 07:00 the next morning, while drinking water was still provided.

### Morris Water Maze

The maze consisted of a circular tank (150 cm in diameter, 30 cm in height) placed on a steel frame. The tank was filled with water (21–22°C, depth of 25 cm). A cylindrical platform (10 cm in diameter and 24 cm in height) was fixed in the center of a quadrant in the pool to allow the mouse to escape. The platform was 1 cm below the surface of the water. The periphery of the tank was surrounded by a white curtain, forming a cylindrical shape with a diameter of 3.5 m. Three black conspicuous markers (circles, squares, and triangles) were suspended equidistantly inside the curtain, 150 cm above the ground. A camera system was installed directly above the tank to record the performance of the mouse in the experimental task. In the positioning navigation (learning) phase, the mice were placed on the platform for 30 s on the first day. Then, and in subsequent days, the mice were randomly placed into the water from different quadrants (except for the quadrant of the platform) facing the pool wall. The mice were allowed to swim for 60 s to find the escape platform, and were allowed to rest on the platform for 30 s if they could not find the platform within the 60 s. They were then put back in their cages. The test was performed four times daily with 15-min intervals for 7 days. During the probe trial (to test the memory, on the last day of learning phase), the platform was withdrawn 2 h after the positioning navigation experiment, and the mouse was placed into the water from the opposite quadrant of the target quadrant and allowed to probe the pool for 60 s. Because the distance swam can better reflect the learning ability of aged and elderly mice in the learning phase (Wang et al., 2010), the average swimming distance was used as the learning ability, and the percent distance swam in the target quadrant was used as the memory performance. The distance swam was recorded using ANY-maze software (Stoelting, United States).

### Tissue Preparation

Approximately 15 days after the behavioral test, the mice were sacrificed by cervical dislocation, decapitated, and their brains quickly removed from the skull. The brains were bisected in the midsagittal plane on dry ice. The left hippocampus was separated from the left hemisphere and stored at −80°C for western blotting. The right hemisphere was fixed in 4% paraformaldehyde at 4°C for 3 days and paraffin-embedded into blocks for hippocampal immunohistochemistry and RNAscope.

### Immunohistochemistry

Paraffin-embedded sections of fixed brains (3 μm thick, in the coronal plane) were prepared on a Leica Microtome (Leica RM 2135, Germany). The hippocampal sections were deparaffinized and rehydrated through a series of xylene and ethanol washes. Antigen retrieval was performed by heating the samples in sodium citrate buffer (0.01 mol/L, pH 6.0) for 20 min in a microwave. Sections were treated with a 0.4% Triton X-100, H_2_O_2_, and 5% bovine serum albumin solution to minimize non-specific binding, and then incubated with anti-Syt1 (1:800; S2177, Sigma, United States) and anti-Arc (1:100; Proteintech, United States) primary antibodies overnight at 4°C. The next day, the sections were rewarmed for 45 min at 37°C, washed three times with PBS (Zsbio, ZLI-9062), incubated with a secondary antibody (biotin-labeled goat anti-rabbit IgG) for 20 min at 37°C, and then treated with a streptavidin-biotin-peroxidase complex (Zhongshan Golden Bridge Biotechnology, Beijing, China) at 37°C for 30 min. Sections that were not incubated with the primary antibody served as negative controls. Finally, the sections were washed three times with PBS, stained with diaminobenzidine (Zsbio, ZLI-9018) at room temperature until coloration became visible, and then quickly washed with tap water to stop color development. Two sections from each animal were stained for each protein. All slices were mounted with neutral gum.

Images of the whole hippocampus (4 × 10) and subfields (20 × 10) were acquired using a digital scanner (Panoramic MIDI). Images of three hippocampal subregions (cornu ammonis [CA]1, CA3, and dentate gyrus [DG]) were obtained.

### Western Blotting

For protein isolation, the left hippocampus was added to a lysis buffer (RIPA buffer, Haiji Biotechnology Co., Ltd., China), disrupted by ultrasonication, and centrifuged at 4°C for 15 min (13,000 rpm). The supernatant was taken as the extracted protein. Protein concentration was measured using a bicinchoninic acid assay kit (Pierce Biotechnology, United States). The protein was solubilized into an equal concentration and added (4:1) to 5× protein electrophoresis loading buffer, mixed, and boiled (100°C, 10 min). The samples were then applied to a 10% SDS-polyacrylamide gel. Beta-actin (TA-09; Zhongshan Golden Bridge Bio-technology) was used as an internal standard. After cooling to room temperature, the protein samples were applied to polyacrylamide gels. Each gel contained hippocampal protein samples and a pre-stained molecular weight marker (Thermo Fisher, United States). The voltage used for the concentrated gel was 80 V for 30 min, and 120 V for 1 h for separating gels. After electrophoresis, the proteins were transferred to polyvinylidene fluoride membranes (Millipore, United States). The membranes were first blocked with 5% skimmed milk in TBS for 2 h at room temperature, and then incubated with rabbit anti-Arc polyclonal (16290-1-AP; Proteintech) and rabbit anti-Syt1 (S2177; Sigma) antibodies overnight at 4°C. After washing with TBS containing 0.1% Tween 20 (TBS-T; Solarbio, T8220; 3 × 10 min), the membranes were incubated with horse radish peroxidase (HRP)-conjugated anti-rabbit IgG (Zhongshan-Golden Bridge Bio-technology; 1/10000) for 2 h at room temperature. Then, the membranes were rinsed with PBS-T (3 × 10 min) and the immunoreactive protein bands were visualized using an enhanced chemiluminescent ECL reagent (Thermo Fisher, United States). Each antibody revealed a single immunoreactive band corresponding to Arc (45 kDa), beta-actin (43 kDa), or Syt1 (47 kDa). Densitometric quantification of band intensities was performed within the range of linear exposure of the film using Image-Pro Plus 6.0 software (Media Cybernetics, United States). Duplicate samples were averaged for each subject. To control for equal loading, ratios of the optical density for the antibody of interest to the optical density of the antibody directed against beta-actin was calculated for each sample.

### RNAscope Assay for mRNA Detection

#### Experimental Procedure

For formalin-fixed, paraffin-embedded (FFPE) tissue, 5-μm thick sections were deparaffinized in xylene and then dehydrated using an ethanol series. A HybEZ hybridization oven was heated to 40°C and a humidity control tray containing distilled water was placed in the oven. Fresh 1× RNAscope Target Retrieval Reagent (ACD, 322000) was prepared and heated to boiling for use. A slide was placed on the slide holder and 5–8 drops of RNAscope H_2_O_2_ were added to the slide, followed by incubation for 10 min at room temperature. Then, the H_2_O_2_ was removed and the slide washed three times with distilled water. The slide was then immersed in 1× RNAscope Target Retrieval Reagent for 15–30 min, washed three times with distilled water, transferred to 100% ethanol for 3 min, and dried at room temperature. Subsequently, a hydrophobic circle was drawn three times around each slice with an Immedge pen and left to dry for 1 min at room temperature. The slide was placed on the holder and five drops of RNAscope protease Plus reagent (ACD, 322381) was added to completely cover the slide. The slide was placed in the HybEZ hybridization oven and incubated at 40°C for 30 min. Then, excess liquid was removed and the slide washed five times with distilled water. Excess liquid was removed and five drops of either the *Arc* (ACD, 316911) or *Syt1* (ACD, 491831) mRNA probe mixture was added to the slide, which was placed in the HybEZ hybridization oven, followed by incubation for 2 h at 40°C. The slide was then washed with 1× wash buffer (2 × 1 min) at room temperature. The liquid on the slide was removed and 4–6 drops of RNAscope multichannel fluorescent second-generation AMP1 (contained in the Multiplex Fluorescent Reagent Kit) were added dropwise onto the slide, which was then incubated at 40°C for 30 min, followed by washing with 1× wash buffer for 2 min at room temperature. AMP2 was added dropwise using the same procedure, followed by incubation at 40°C for 30 min. AMP3 was added dropwise, followed by incubation at 40°C for 15 min. The slide was washed with 1× wash buffer for 2 min, excessive liquid was removed, and 4–6 drops of HRP-C1 were added to completely cover the entire section, following which the slide was placed in the HybEZ hybridization oven and incubated for 15 min at 40°C. Then, the slide was rinsed with fresh 1× wash buffer for 2 min at room temperature. After removing excess liquid, 150–200 μL of TSA plus fluorescent dye was added dropwise to the slide, which was then placed in the HybEZ hybridization oven, incubated at 40°C for 30 min, and rinsed with 1× wash buffer for 2 min. After removing the excess liquid, 4–6 drops of RNAscope multichannel fluorescent second-generation HRP blocker was added to the slide, which was again placed in the HybEZ hybridization oven, incubated for 15 min at 40°C, and rinsed with 1× wash buffer for 2 min. After removing excess liquid, four drops of DAPI were added to the slide which was then incubated for 30 s at room temperature. After removing the DAPI, 1–2 drops of Prolong Gold antiquenching seal was added, and the slide was dried for 30 min in the dark overnight. Slides were stored in the dark at 4°C. Brain sections were observed under a fluorescence microscope.

#### Morphometric Analysis and Quantification

Images were acquired using an Olympus IX71 fluorescent microscope (Olympus, Tokyo, Japan) equipped with a PXL37 CCD camera (Photometrics, Tucson, AZ, United States). For multiplex RNAscope staining and for studies with FFPE tissue specimens, images were acquired using a Zeiss Axioplan M1 microscope (Carl Zeiss Micro Imaging, Göttingen, Germany) equipped with a CRi Nuance multispectral imaging system (Caliper Life Sciences, Cambridge, MA, United States). Overlapping signals from different fluorophores were separated by comparing composite signals against a reference spectral library generated with samples stained with a single color. RNAscope hybridization fluorescence was imaged using a ×40 objective on a laser-scanning confocal microscope (Zeiss LSM700 or LSM780). During each imaging session, both *Arc* and *Syt1* slices that were processed simultaneously in the same hybridization experiment were imaged. The microscope settings were fixed in each imaging session. Images were obtained for six brains per group (at least 6 sections/mouse) and Image J software was used to calculate the mean fluorescence intensity in the three subregions of the hippocampus, i.e., the CA1, CA3, and DG.

### Statistical Analysis

All results were expressed as means ± standard error of the mean (SEM) for the parametric data or as 50th (25th/75th) quartiles for non-parametric data. For the performances in the learning phase of the Morris water maze (MWM) task, the data were analyzed using repeated measures analysis of variance (rm-ANOVAs) with day, age, or treatment as independent variables. For comparison of the results among the different groups, *post hoc* analysis was performed using Fisher’s least-significant difference test. The parametric data were analyzed using two-way ANOVA with age or treatment as independent variables. For non-normally distributed data, the Kruskal–Wallis H test was used, followed by an extended *t*-test for pair-wise analysis. Pearson’s correlation test was used to analyze the correlations between the relative Arc and Syt1 protein/mRNA levels in hippocampal subregions and the performance in all trials of the learning or memory phase in the MWM. Significance was assumed at *P* < 0.05. All the analyses were conducted using SPSS 21.0 for Windows.

## Results

### Performances in the MWM

#### Learning Phase

##### Age effects

**FIGURE 2:**
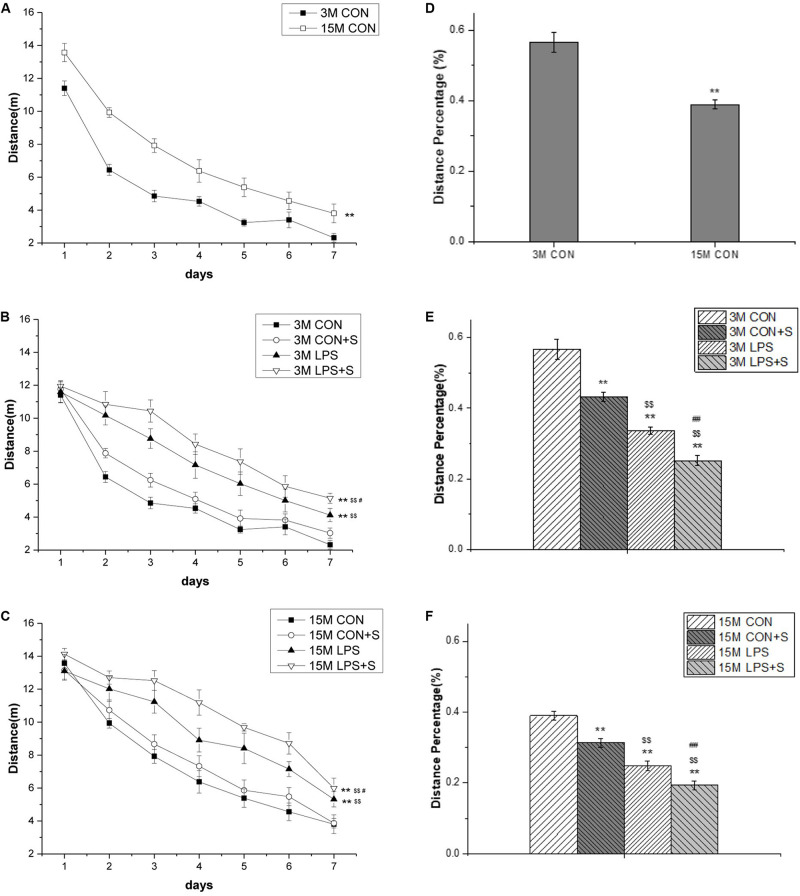
The distance swam in the learning phase and the percent swimming distance in the target quadrant in the memory phase of the MWM test in CD-1 mice. **(A)** Distance swam in the learning phase at different ages in the control groups. **(B)** Distance swam in the learning phase by 3-month-old (3 M) and **(C)** 15-month-old (15 M) CD-1 mice in the different treatment groups. **(D)** Percent distance swam in the target quadrant by mice of different ages from the control, untreated groups. **(E)** Comparisons among the different treatment groups of percent distance swam in the target quadrant by 3 M **(F)** and 15 M CD-1 mice. Error bars = SEM. *P < 0.05, **P < 0.01 compared with the control group; ^$^*P* < 0.05, ^$$^*P* < 0.01 compared with the CON + S group; ^#^*P* < 0.05, ^##^*P* < 0.01 compared with the LPS group. CON, untreated control group; LPS, lipopolysaccharide treatment group; S, group of mice exposed to stress; MWM, Morris water maze.

The distance swam declined progressively for all the control mice (*F*_[6,60]_ = 173.18, *P* < 0.001), indicating that these mice were able to learn the task. There was a significant effect of age and interaction of ages × days on distance swam (*F*_[1,10]_ = 24.67, *P* = 0.001; *F*_[6,60]_ = 2.89, *P* = 0.015). *Post hoc* analysis indicated that 15-month-old mice from the CON group swam significantly longer distances than the 3-month-old mice from the same group (*P* < 0.01; Figure 2A). Similarly, 15-month-old mice swam significantly longer distances than 3-month-old mice in all the treatment groups (CON + S, LPS, and LPS + S) (*Ps* < 0.01, *n* = 6 per group; Supplementary Figures S1A–C).

##### Treatment effects

For the 3-month-old mice, there were significant differences in swimming distances among the different treatment groups (*F*_[3,23]_ = 26.40, *P* < 0.001). The *post hoc* analysis showed that mice from the LPS and LPS + S groups swam significantly longer distances than the mice from the CON group (*Ps* < 0.01); however, the differences were only marginal compared with the CON + S group (*P* = 0.075). Meanwhile, there were significant differences between the LPS and CON + S groups (*P* = 0.001) and between the LPS and LPS + S groups (*P* = 0.025; Figure 2B). For the 15-month-old mice, significant differences in swimming distances were found among the different treatment groups (*F*_[3,23]_ = 15.08, *P* < 0.001). Furthermore, the learning swimming distances in the LPS (*P* = 0.001) and LPS + S (*P* < 0.001) groups were significantly longer than those in the CON and CON + S groups; the difference between the CON and CON + S groups were not significant (*P* = 0.374). Moreover, mice from the LPS + S group exhibited significantly longer swimming distances than those from the LPS group (*P* < 0.05; Figure 2C).

#### Memory Phase

##### Age effects

The 15-month-old mice in the CON group had a significantly lower percentage of distance swam in the target quadrant than the 3-month-old mice from the same group (*t* = 5.64; *P* < 0.01; Figure 2D), as did those in the CON + S, LPS, and LPS + S groups (*Ps* < 0.05, *n* = 6 per group; Supplementary Figures S1D–F).

##### Treatment effects

There were significant differences in the percent distance swam among the four groups at both 3 months (*F*_[3,23]_ = 55.09, *P* < 0.001) and 15 months of age (*F*_[3,23]_ = 45.32, *P* < 0.001). The *post hoc* analyses showed that the distance percentage was significantly smaller in the CON + S, LPS, and LPS + S groups than in the CON group (*n* = 6 per group, *Ps* < 0.01), irrespective of age. Specifically, the distance percentage in the LPS group was smaller than in the CON + S group (*Ps* < 0.01), but larger than in the LPS + S group (*Ps* < 0.01; see Figures 2E,F).

### Levels of Arc and Syt1 in the Hippocampus

#### Immunohistochemical Analysis

**FIGURE 3:**
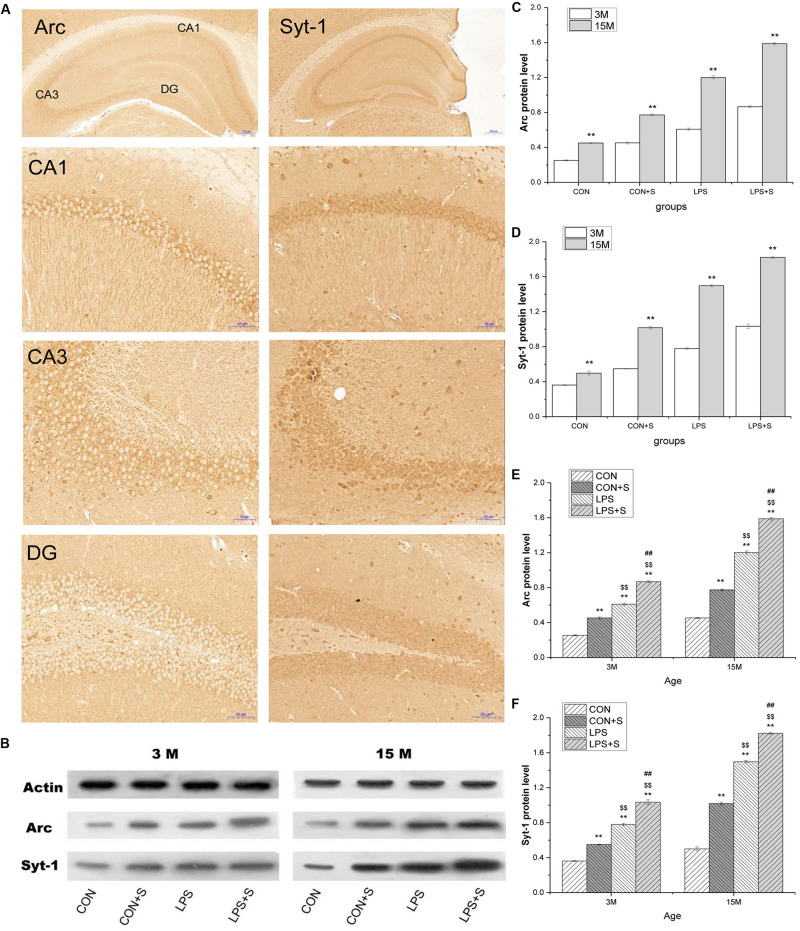
The hippocampal protein levels of Arc and Syt1 in CD-1 mice. **(A)** Representative photomicrographs of Arc and Syt1 protein immunolabeling in the dorsal hippocampus and its subfields in 15-month-old (15 M) LPS + S-treated mice (top row is the dorsal hippocampus, low magnification; second to fourth rows represent different subregions, high magnification). **(B)** Representative Arc and Syt1 immunoreactive bands in the hippocampi of mice in the different treatment groups at different ages. **(C,E)** Arc and **(D,F)** Syt1 protein levels in the hippocampi of mice of different ages in the different treatment groups. Scale bar = 200 μm at low magnification and 50 μm at high magnification. Error bars = SEM. *P < 0.05, **P < 0.01 compared with the control group; *^$^P* < 0.05, *^$$^P* < 0.01 compared with the CON + S group; *^#^P* < 0.05, *^##^P* < 0.01 compared with the LPS group.

Representative photomicrographs of immunolabeled Arc and Syt1 proteins in hippocampal subfields (CA1, CA3, and DG) of 15-month-old mice from the LPS + S group are shown in Figure 3A. Punctate staining is distributed throughout every layer of the CA1, CA3, and DG subregions. Moreover, the distribution of immunoreactivity for both proteins from the different treatment groups differed between older (15 months old) and younger (3 months old) mice, but was more evident in 15-month-old mice (*n* = 6 per group; Supplementary Figure S2). The immunoreactivity in both 3- and 15-month-old mice was greater with than without embryonic exposure to inflammation. Meanwhile, stress exposure in adolescence increased the immunoreactivity in the mice also exposed to embryonic inflammation (Supplementary Figure S2).

#### Western Blotting Analysis

The protein levels of Arc and Syt1 were significantly higher in 15-month-old mice from the CON group than in 3-month-old mice from the same group (Figures 3B–D). Interestingly, the different treatments significantly affected the hippocampal levels of Arc and Syt1, both in the young (*n* = 6 per group, *F*_[3,23]_ = 626.78, 371.98; *Ps* < 0.001) and older mice (*n* = 6 per group, *F*_[3,23]_ = 1460.14, 1357.49; *Ps* < 0.001). Among the 3-month-old mice, those in the CON + S, LPS, and LPS + S treatment groups exhibited significantly higher Arc and Syt1 protein levels than the 3-month-old mice from the CON group (*Ps* < 0.001). The Arc and Syt1 protein levels in the LPS treatment group were significantly higher than those in the CON + S group (*n* = 6 per group, *Ps* < 0.001), but lower than those in the LPS + S group (*Ps* < 0.001). Similarly, among the 15-month-old mice, the Arc and Syt1 protein levels were significantly higher in the three treatment groups than in the CON group (*Ps* < 0.001). However, the levels of both proteins in the LPS group were significantly higher than those in the CON + S group (*Ps* < 0.01), but significantly lower than those in the LPS + S group (*Ps* < 0.01; Figures 3E,F).

### *Arc and Syt1* mRNA Levels in Different Hippocampal Subregions

**FIGURE 4:**
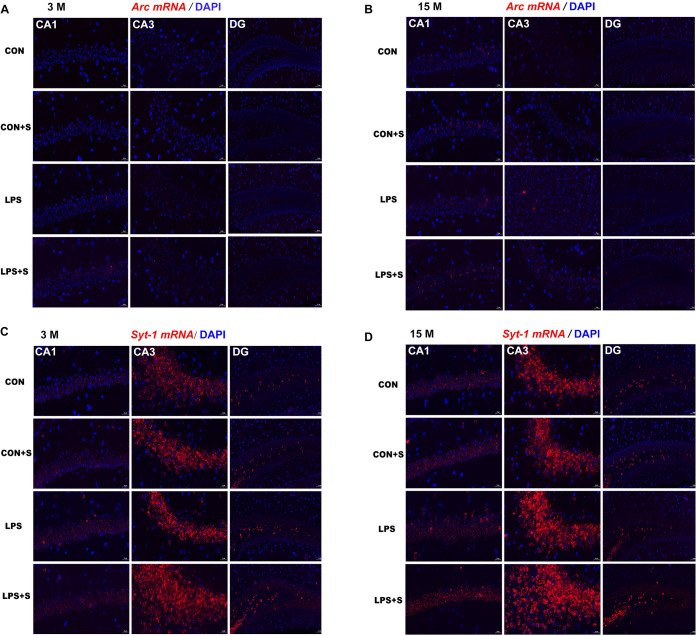
Representative photomicrographs of *Arc* and *Syt1* mRNA levels in different hippocampal subregions in CD-1 mice of different ages and under different treatments. Hippocampal *Arc* **(A,B)** and *Syt1* **(C,D)** mRNA levels representing three subregions of the dorsal hippocampus in 3-month-old (3 M) **(A,C)** and 15-month-old (15 M) **(B,D)** mice under different treatments. Scale bar = 20 μm in the CA1 and CA3, 50 μm in the DG. CA, cornu ammonis; DG, dentate gyrus.

*In situ* hybridization fluorescent immunostaining showed that *Arc* and *Syt1* transcripts were primarily localized in the pyramidal cell layer (Figure 4). In 3-month-old mice, a significant intergroup difference was found for *Arc* transcripts in the CA1 (*F*_[3,23]_ = 38.71, *P* < 0.001) and CA3 (*F*_[3,23]_ = 5.81, *P* < 0.01) subregions, and for *Syt1* mRNA in the CA3 (*F*_[3,23]_ = 124.261, *P* < 0.001) subregion. Similarly, in 15-month-old mice, significant intergroup differences in *Arc* and *Syt1* mRNA levels were also found in the CA1 (*F*_[3,23]_ = 13.98, 20.59; *Ps* < 0.001) and CA3 (*F*_[3,23]_ = 21.42, 142.11; *Ps* < 0.001) subregions. In addition, for both mRNAs, significant differences were found in the corresponding hippocampal subregions for each treatment group in the 15-month-old mice compared with the 3-month-old mice (*n* = 6 per group; Supplementary Figures S3, S4).

#### *Post hoc* Analysis

**FIGURE 5:**
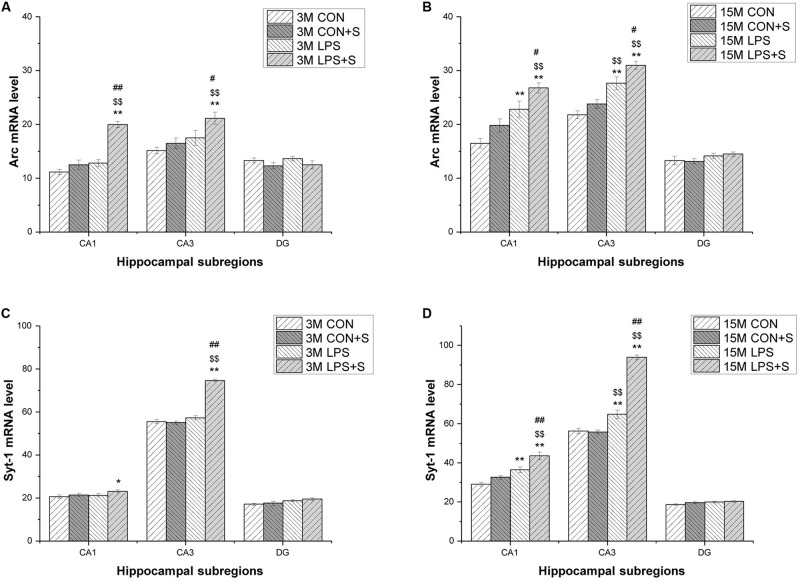
The levels of *Arc* and *Syt1* mRNA in different hippocampal subregions in CD-1 mice. **(A,B)** *Arc* mRNA, and **(C,D)** *Syt1* mRNA levels in different hippocampal subregions (CA1, CA3, and DG) in the different treatment groups at 3 months (3 M) **(A,C)** and 15 months (15 M) **(B,D)** of age. Error bars = SEM. *P < 0.05, **P < 0.01 compared with the control group; *^$^P* < 0.05, *^$$^P* < 0.01 compared with the CON + S group; *^#^P* < 0.05, *^##^P* < 0.01 compared with the LPS group. CA, cornu ammonis; DG, dentate gyrus; CON, untreated control group; LPS, lipopolysaccharide treatment group; S, group of mice exposed to stress.

In the 3-month-old mice, *Arc* and *Syt1* transcript levels were both significantly increased in all the treatment groups (*n* = 6 per group, *Ps* < 0.05) compared with those in the CON group. Mice in the LPS + S group presented significantly higher *Arc* mRNA levels than the LPS, CON + S, and CON groups in the CA1 (*Ps* < 0.001) and CA3 (*Ps* < 0.05) subregions, as did *Syt1* mRNA levels in the CA3 (*Ps* < 0.001). In the CA1 and DG, significant differences in *Syt1* mRNA levels were found only between the LPS + S and CON groups (*P* = 0.039, *P* = 0.021). The 15-month-old mice from the LPS and LPS + S groups showed significantly increased *Arc* and *Syt1* mRNA levels in the CA1 and CA3 subregions (*n* = 6 per group, *Ps* < 0.01) compared with the CON group, with similar changes observed in the LPS + S group relative to the LPS and CON + S groups (*Ps* < 0.05). The *Arc* and *Syt1* mRNA levels in the CA3 subregion were higher in the LPS group than in the CON + S group (*Ps* < 0.01). However, in the DG, significant differences in *Syt1* mRNA levels were found only between the LPS + S and CON groups (*P* = 0.024; Figure 5).

### Correlations Between Performance in the MWM Test and Arc and Syt1 Expression Levels

#### Correlations Between Performance and Protein Levels

In the 3-month-old mice, hippocampal Arc and Syt1 levels showed a significant positive correlation with the learning swimming distance (*r* = 0.908, 0.925; *Ps* < 0.01) and a negative correlation with the percent of distance swam in the target quadrant (*Ps* < 0.01) for the four groups combined. For each group, the learning swimming distance was positively correlated with Arc levels in the LPS + S group (*P* = 0.027) and with Syt1 levels in the LPS and LPS + S groups (*Ps* < 0.05), and the memory distance percentage was negatively correlated with Arc (*r* = −0.940, 0.890; *P* = 0.005, 0.017) and Syt1 levels (*r* = −0.976, −0.914; *P* = 0.001, 0.011) in the LPS and LPS + S groups. In the 15-month-old mice, the levels of Arc and Syt1 were also positively correlated with the learning swimming distance (*r* = 0.855, 0.837; *Ps* < 0.001), and negatively correlated with the percent distance swam in the target quadrant (*Ps* < 0.05) for all the groups combined. For individual treatments, there were negative correlations between performance and protein levels in almost four groups (*Ps* < 0.05; Table 1).

**TABLE 1:** The correlations between the performance in the MWM test and hippocampal synaptic protein levels.

Cognitive phase	Age	Group	Synaptic protein	
			
			Arc	Syt1
			*r (p)*	*r (p)*
Swimming	3 months	CON	0.784 (0.065)	0.631 (0.179)
distance		CON + S	0.609 (0.200)	0.802 (0.055)
		LPS	0.805 (0.053)	0.838(0.037)*
		LPS + S	0.862(0.027)*	0.872(0.024)*
	15 months	CON	0.876(0.022)*	0.974(0.001)**
		CON + S	0.842(0.036)*	0.874(0.023)*
		LPS	0.900(0.015)*	0.897(0.015)*
		LPS + S	0.904(0.013)*	0.981(0.001)**
Distance	3 months	CON	−0.013(0.98)	−0.127(0.810)
percentage		CON + S	−0.635(0.175)	−0.608(0.200)
in target		LPS	−0.940(0.005)**	−0.976(0.001)**
quadrant		LPS + S	−0.890(0.017)*	−0.914(0.011)*
	15 months	CON	−0.929(0.007)**	−0.954(0.003)**
		CON + S	−0.928(0.008)**	−0.946(0.004)**
		LPS	−0.990(0.000)**	−0.917(0.010)*
		LPS + S	−0.934(0.006)**	−0.908(0.012)*

*P < 0.05; **P < 0.01. CON, untreated control group; LPS, lipopolysaccharide treatment group; S, group of mice exposed to stress.

#### Correlations Between Performance and mRNA Levels

As shown in Table 2, in the 3-month-old mice, the swimming distances in the learning phase showed significant positive correlations with the levels of *Arc* mRNA in the CA1 of mice from the LPS and LPS + S groups (*Ps* < 0.05), and in the CA3 of mice from the CON + S group (*r* = 0.871; *P* = 0.024). There were significant positive correlations for *Syt1* mRNA levels in the CA3 of mice from the LPS (*r* = 0.884, *P* = 0.019) and LPS + S groups (*r* = 0.882, *P* = 0.02). The memory distance percentage was negatively correlated with the CA1 levels of *Arc* mRNA in the LPS + S and LPS groups (*Ps* < 0.05), CA1 levels of *Syt1* mRNA in the LPS + S, LPS, and CON + S groups (*Ps* < 0.05), and CA3 levels of *Syt1* mRNA in the LPS + S group (*P* < 0.01).

**TABLE 2:** The correlations between the performance in the MWM test and hippocampal mRNA levels.

Cognitive phase	Age	Group	*Arc* mRNA			*Syt1* mRNA		
			CA1	CA3	DG	CA1	CA3	DG
			*r (p)*	*r (p)*	*r (p)*	*r (p)*	*r (p)*	*r (p)*
Learning	3 months	CON	0.630 (0.18)	0.401 (0.431)	−0.018(0.973)	0.605 (0.203)	0.547 (0.261)	−0.338(0.513)
swimming		CON + S	0.514 (0.297)	0.871(0.024)*	−0.186(0.724)	0.418 (0.410)	0.410 (0.420)	−0.232(0.658)
distance		LPS	0.922(0.009)**	0.357 (0.487)	−0.616(0.193)	0.884(0.019)*	0.763 (0.077)	−0.096(0.857)
		LPS + S	0.901(0.014)*	0.367 (0.474)	−0.017(0.975)	0.789 (0.062)	0.882(0.020)*	−0.398(0.435)
	15 months	CON	−0.176(0.738)	−0.630(0.180)	−0.567(0.241)	0.416 (0.412)	0.601 (0.198)	0.778 (0.069)
		CON + S	0.953(0.003)**	0.981(0.001)**	−0.049(0.926)	0.982(0.000)**	0.756 (0.082)	0.321 (0.535)
		LPS	0.878(0.022)*	0.800 (0.056)	−0.450(0.370)	0.879(0.021)*	0.938(0.006)**	−0.322(0.534)
		LPS + S	0.875(0.022)*	0.825(0.043)*	0.637 (0.174)	0.925(0.008)**	0.897(0.015)*	0.496 (0.317)
Distance	3 months	CON	−0.030(0.955)	−0.543(0.266)	−0.545(0.264)	0.438 (0.385)	0.480 (0.335)	0.579 (0.228)
percentage		CON + S	−0.633(0.178)	−0.102(0.847)	0.329 (0.524)	−0.813(0.049)*	0.673 (0.143)	−0.243(0.643)
in target		LPS	−0.867(0.025)*	−0.776(0.07)	0.309 (0.551)	−0.851(0.032)*	−0.336(0.515)	0.473 (0.343)
quadrant		LPS + S	−0.975(0.001)**	−0.434(0.39)	−0.258(0.621)	−0.903(0.014)*	−0.973(0.001)**	−0.124(0.816)
	15 months	CON	0.163 (0.758)	0.583 (0.224)	0.349 (0.498)	−0.533(0.288)	−0.790(0.062)	−0.707(0.117)
		CON + S	−0.661(0.153)	−0.817(0.047)*	−0.324(0.531)	−0.783(0.066)	−0.992(0.000)**	−0.181(0.731)
		LPS	−0.988(0.000)**	−0.933(0.007)**	0.096 (0.856)	−0.967(0.002)**	−0.816(0.048)*	−0.164(0.756)
		LPS + S	−0.918(0.01)**	−0.849(0.032)*	−0.315(0.544)	−0.966(0.002)**	−0.952(0.003)**	0.045 (0.932)

*P < 0.05; **P < 0.01. CA, cornu ammonis; DG, dentate gyrus; CON, untreated control group; LPS, lipopolysaccharide treatment group; S, group of mice exposed to stress.

In the 15-month-old mice, a significant positive correlation was found between *Arc* mRNA levels and the learning swimming distance in the CA1 of the LPS + S, LPS, and CON + S groups (*Ps* < 0.05) and CA3 of the LPS + S and CON + S groups (*Ps* < 0.05). Additionally, a significant negative correlation was found between *Arc* mRNA levels and the memory distance percentage in the CA1 of the LPS + S and LPS groups (*Ps* < 0.05) and CA3 of the LPS + S, LPS, and CON + S groups (*Ps* < 0.05). A significant positive correlation was found between *Syt1* mRNA levels and the learning swimming distance in the CA1 of the LPS + S, LPS, and CON + S groups (*Ps* < 0.05) and the CA3 of the LPS + S and LPS groups (*Ps* < 0.05). A significant negative correlation was also found between *Syt1* mRNA levels and the memory distance percentage in the CA1 of the LPS + S and LPS groups (*Ps* < 0.05) and CA3 of the LPS + S, LPS, and CON + S groups (*Ps* < 0.05; Table 2).

## Discussion

Embryonic exposure to inflammation resulting from infections in pregnant mothers may be an important mechanism underlying age-related behavioral impairment, especially AISLM, in both humans and rodents. Several studies have demonstrated that there is a gender-dependent effect on memory in normal aging as well as on prenatal exposure to different insults; however, the results have not been consistent. For instance, we have previously shown that AISLM exists in older female, but not male, Kunming mice (Chen et al., 2004). Hernandez et al. (2020) demonstrated that there are no significant differences between the sexes in age-related cognitive impairment (including spatial and working memory) in rats. Wang et al. (2019) showed that prenatal exposure to inflammation leads to increased anxiety-related behavior in adult male offspring when compared with adult female offspring. Fernández de Cossío et al. (2017) also reported a male-specific effect for intrauterine LPS exposure on synaptic pruning in the hippocampus of adult offspring, while the observed LPS-induced anxiety-related behavior deficits did not differ between the sexes. Our recent study showed no difference between female and male offspring in the effect of maternal prenatal LPS exposure on AILSM in CD-1 mice (Li X. W. et al., 2016). In the present study, we only selected male offspring, and the results indicated that stress in male offspring during adolescence will accelerate the AISLM caused by exposure to inflammation during the embryonic stage. Moreover, we also demonstrated that there was an age-related increase in the expression of Arc and Syt1 at both the protein and mRNA levels in the dorsal hippocampus, which could be further increased following embryonic exposure to an inflammatory environment, with or without exposure to stress during adolescence. Interestingly, these altered expression levels of synaptic proteins might be linked to impaired spatial learning and memory caused by different treatments in embryos and/or adolescents.

### The Effects of Prenatal Exposure to Inflammation Coupled With Stress Exposure in Adolescence on AISLM in Midlife

Several studies have confirmed that exposure to adverse environments in early life may lead to permanent changes in key organs or tissues during the developmental “window” period; in turn, these changes may trigger preset procedural and structural changes in relevant brain regions related to processes such as memory formation and stress response (Batinić et al., 2016; Weinstock, 2016). Our results indicated that learning and memory ability decreases gradually with age. Moreover, mice that were exposed to inflammatory environments as embryos showed a significantly worse performance in the MWM test than unexposed mice, regardless of age (3 or 15 months). Studies have shown that stressors presented in the late prenatal or early postnatal periods, the periods when the formation of brain circuits associated with early development occurs, have long-term effects on the behavior of offspring (Yang et al., 2007). In this study, all the mice from the LPS, LPS + S, and CON + S treatment groups exhibited worse memory performance than the CON group. Furthermore, mice from the LPS and LPS + S treatment groups had worse memory performance than those in the CON + S group at both ages. These results suggested that exposure to an inflammatory environment during the embryonic period is an important accelerator of AISLM, and exposure to stress during adolescence may further aggravate this effect.

### The Age-Related Changes in Hippocampal Arc and Syt1 Expression

Arc is a postsynaptic protein critical for memory consolidation, especially that of spatial memory (Plath et al., 2006; Ramirez-Amaya et al., 2013). However, relatively few studies have reported on age-related changes in *Arc* gene expression in the brain, and the reported results have been inconsistent. The hippocampus from aged (24–27 months) male Fischer-344 rats has a similar number of *Arc* mRNA-expressing pyramidal cells in the CA1 and CA3 regions compared with that in the adult (10–12 months) hippocampus, as labeled by fluorescence *in situ* hybridization (Marrone et al., 2012). However, the hippocampus of aged mice (24 months old) has reduced levels of *Arc* mRNA, as detected with RT-RNA and *in situ* hybridization, in C57BL/6J mice relative to that in young (6 months old) mice (Qiu et al., 2016). In contrast, aged (24 months of age) Long-Evans male rats have increased basal Arc protein levels in the CA1 field of the hippocampus when compared with young rats (Fletcher et al., 2014). Our results suggested that in the hippocampi of aged CD-1 mice, Arc transcription and translation are both upregulated, in agreement with previous results (Fletcher et al., 2014; Maurer et al., 2017).

Syt1 plays a modulatory role in endocytosis, and has recently been implicated in the calcium-sensitive trafficking of postsynaptic AMPARs to facilitate long-term potentiation (Hussain et al., 2017; Wu et al., 2017). Therefore, Syt1 and Arc can synergistically regulate the number of AMPARs on the postsynaptic membrane to regulate synaptic plasticity and, consequently, learning and memory. In the current study, the hippocampal Syt1 and *Syt1* mRNA levels were significantly higher in 15-month-old mice than in 3-month-old mice from the CON group. These findings about Syt1 protein levels were consistent with the results from CD-1 and SAMP8 mice (Tong et al., 2015; Li X. W. et al., 2016; Li X. Y. et al., 2016), but *Syt1* mRNA levels were inconsistent with the result from SAMP8 mice (Chen et al., 2007). This discrepancy may be due to different strains of rodents and experimental manipulation.

### The Effect of Prenatal Inflammatory Insult Coupled With Stress Exposure in Adolescence on Hippocampal *Arc and Syt1* Gene Expression

To date, no study has investigated the effect of maternal inflammatory insult during gestation on the transcript levels of synaptic protein-related genes in the offspring, or the effects of prenatal exposure to inflammation coupled with stress exposure in adolescence on the expressions of synaptic protein-related genes in offspring of different ages. In the current study, aged mice had higher levels of Arc and Syt1 at both the protein (detected by western blotting) and mRNA (detected by RNAscope) levels in the whole hippocampus than young mice. Interestingly, different treatments significantly affected hippocampal Arc and Syt1 levels, regardless of age. These results suggested that prenatal exposure to inflammation can enhance hippocampal expression of the *Arc* and *Syt1* genes in the offspring, irrespective of age, and additional exposure to stress in adolescence can further increase these levels. Notably, although exposure to stress (without LPS treatment) during adolescence could also significantly increase the expression levels of the *Arc* and *Syt1* genes in the hippocampus from youth to midlife, the extent of this effect was significantly less than the effect of prenatal exposure to inflammation. The fact that the aged mice had higher hippocampal expression levels of the *Arc* and *Syt1* genes than the young mice, and that the above-mentioned treatments elicited different effects, indicated that a prenatal inflammatory insult can aggravate the age-related increase in the hippocampal levels of Arc and Syt1, and exposure to stress during adolescence can further exacerbate this change in the levels of synaptic proteins.

### The Correlation Between Cognitive Performance and *Arc* and *Syt1* Gene Expression

Memory loss due to aging is accompanied by a downregulation of AMPARs that mediate fast excitatory synaptic transmission. The dynamic regulation of AMPARs is crucial for supporting basal synaptic transmission and plasticity. Arc-mediated regulation of the endocytic pathway modulates the basal levels of AMPARs and increased Arc expression leads to reduced AMPA transmission through GluR2/3 removal. To date, studies investigating the correlation between Arc expression and memory have shown inconsistent results. For example, Arc protein expression is decreased in the hippocampus during memory loss in C57/BL6 mice (Gautam et al., 2016), while a decline in *Arc* gene expression might be associated with age-dependent memory decline in male Swiss albino mice (Singh and Thakur, 2018). Aged Long-Evans male rats with memory impairment have increased basal Arc protein levels in the CA1 field of the hippocampus (Fletcher et al., 2014). In addition, cells with increased Arc levels exhibit impaired long-term depression, while chronic Arc overexpression is linked to abnormal spine structure (Kelly and Deadwyler, 2003). Syt1 may be a key regulator of AMPAR insertion in postsynaptic spine membranes (Hussain et al., 2017). Increased Syt1 levels can change synaptic transmission and also have a marked effect on the morphology of neurons (Inoue et al., 2015). In rats, stress can increase hippocampal Syt1 expression, which may be partially responsible for changes in neuronal morphology, biochemistry, and behavior, including learning and memory deficits (Thome et al., 2001). Therefore, we hypothesized that Arc and Syt1 overexpression are likely not to be beneficial for learning and memory.

Behavioral testing can induce stress-related changes in plasticity markers as well as in gene expression (Crawley et al., 1997). To mitigate this potential bias, we allowed 2 weeks of “recovery” from behavioral testing before performing biochemical analysis. In this study, the spatial learning and memory ability of 15-month-old mice decreased significantly and the protein and mRNA levels of Arc and Syt1 increased in parallel when compared with their younger (3 months old) siblings. This suggests that increased hippocampal expression of the *Arc* and *Syt1* genes may be involved in the impaired spatial learning ability and memory resulting from conditions such as aging, stress, and prenatal exposure to inflammation. Indeed, the correlation analysis indicated that performance in the MWM test was significantly correlated with changed Arc and Syt1 protein and mRNA levels for all the mice. Specifically, in the normally aging mice, the learning swimming distance positively, and memory distance percentage negatively, correlated with the hippocampal levels of Arc and Syt1 in 15-month-old mice, but not in 3-month-old mice (Table 1). Interestingly, because prenatal exposure to inflammation or exposure to stress in adolescence affected the cognitive behaviors and hippocampal levels of Arc and Syt1 in the 3- and 15-month-old mice, the hippocampal protein levels of Arc and Syt1 in the treatment groups were positively correlated with the learning swimming distance and negatively correlated with the percent distance swam (Table 1).

Overall, the pattern of correlation between MWM performance and mRNA or protein levels was similar. However, in the untreated controls, no significant correlation was recorded between the MWM performance and *Arc* and *Syt1* mRNA levels, irrespective of age. For the groups undergoing different treatments, learning swimming distance was positively, and memory distance percentage negatively, correlated with the levels of *Arc* and *Syt1* mRNA in the CA1 and CA3, but not DG, hippocampal subregions of the 3-month-old mice (Table 2). Similar correlations were observed in the CA1 and/or CA3 subregions at 15 months of age in all the treatment groups (LPS, LPS + S, and CON + S; Table 2). The above results indicated that only the increased hippocampal expression of the Arc and Syt1 proteins was associated with AISLM. Moreover, increased hippocampal expression of Arc and Syt1 at both the protein and mRNA levels was associated with a decline in spatial learning and memory during “pathological” aging due to prenatal inflammatory insult, whether or not this course was exacerbated by exposure to stress in adolescence.

In conclusion, LPS administration to CD-1 mice during pregnancy can lead to long-lasting enhanced hippocampal expression of the *Arc* and *Syt1* genes in offspring from adolescence onward, which can affect the behavior of the mice. In particular, increased expression of these genes can lead to impaired spatial learning and memory as well as the normal aging process. Moreover, exposure to stress during adolescence may further accelerate the AISLM and enhance the hippocampal expression of the *Arc* and *Syt1* genes. Notably, functional differences exist between the left and right hippocampi of rodents, depending on the demand for short-term or long-term memory (Shipton et al., 2014; Sakaguchi and Sakurai, 2020). Due to experimental limitations, we did not consider the left–right anatomical and functional differences of the rodent hippocampus. However, all the animal experiments were performed using the same standards. Although this study was limited by the specimen number and gender, it did reveal that adverse events during pregnancy might contribute to accelerated aging and AISLM in male offspring. Further research is needed to explore whether similar effects occur in females.

## Data Availability Statement

All datasets generated for this study are included in the article/Supplementary Material.

## Ethics Statement

The animal study was reviewed and approved by the Association of Laboratory Animal Sciences and the Center for Laboratory Animal Sciences at Anhui Medical University.

## Author Contributions

Z-ZZ and Z-QZ conceived and designed the study and performed the western blotting and RNAscope tests. Z-ZZ drafted the manuscript. S-YS, H-HG, and Y-FW performed immunohistochemistry and the behavioral test. LC, LX, and Q-GY participated in the study design and statistical analysis. G-HC and FW revised the manuscript. All authors read and approved the final manuscript.

## Conflict of Interest

The authors declare that the research was conducted in the absence of any commercial or financial relationships that could be construed as a potential conflict of interest.

## References

[B1] Akbarian S. Beeri M. S. Haroutunian V. (2013). Epigenetic determinants of healthy and diseased brain aging and cognition. *JAMA Neurol.* 70 711–778. 10.1001/jamaneurol.2013.1459 23571692 PMC3932340

[B2] Arrode-Brusés G. Brusés J. L. (2012). Maternal immune activation by poly I:C induces expression of cytokines IL-1β and IL-13, chemokine MCP-1 and colony stimulating factor VEGF in fetal mouse brain. *J. Neuroinflam.* 9:83. 10.1186/1742-2094-9-83 22546005 PMC3413576

[B3] Barylko B. Wilkerson J. R. Cavalier S. H. Binns D. D. James N. G. Jameson D. M. (2018). Palmitoylation and membrane binding of Arc/Arg3.1: A potential role in synaptic depression. *Biochemistry* 57 520–524. 10.1021/acs.biochem.7b00959 29264923 PMC10370338

[B4] Batinić B. Santrač A. Divović B. Timić T. Stanković T. Obradović A. L. J. (2016). Lipopolysaccharide exposure during late embryogenesis results in diminished locomotor activity and amphetamine response in females and spatial cognition impairment in males in adult, but not adolescent rat offspring. *Behav. Brain Res.* 299 72–80. 10.1016/j.bbr.2015.11.025 26620494

[B5] Bettio L. E. B. Rajendran L. Gil-Mohapel J. (2017). The effects of aging in the hippocampus and cognitive decline. *Neurosci. Biobehav. Rev.* 79 66–86. 10.1016/j.neubiorev.2017.04.030 28476525

[B6] Cao L. Wang F. Yang Q. G. Jiang W. Wang C. Chen Y. P. (2012). Reduced thyroid hormones with increased hippocampal SNAP-25 and Munc18-1 might involve cognitive impairment during aging. *Behav. Brain Res.* 229 131–137. 10.1016/j.bbr.2012.01.014 22261019

[B7] Chang S. Trimbuch T. Rosenmund C. (2018). Synaptotagmin-1 drives synchronous Ca-triggered fusion by CB-domain-mediated synaptic-vesicle-membrane attachment. *Nat. Neurosci.* 21 33–40. 10.1038/s41593-017-0037-5 29230057 PMC5742540

[B8] Chen G. H. Wang Y. J. Qin S. Yang Q. G. Zhou J. N. Liu R. Y. (2007). Age-related spatial cognitive impairment is correlated with increase of synaptotagmin 1 in dorsal hippocampus in SAMP8 mice. *Neurobiol. Aging* 28 611–618. 10.1016/j.neurobiolaging.2006.03.001 16677738

[B9] Chen G. H. Wang Y. J. Zhang L. Q. Zhou J. N. (2004). Age- and sex-related disturbance in a battery of sensorimotor and cognitive tasks in Kunming mice. *Physiol. Behav.* 83 531–541. 10.1016/j.physbeh.2004.09.012 15581676

[B10] Crawley J. N. Belknap J. K. Collins A. Crabbe J. C. Frankel W. Henderson N. (1997). Behavioral phenotypes of inbred mouse strains: implications and recommendations for molecular studies. *Psychopharmacology* 132 107–124. 10.1007/s002130050327 9266608

[B11] Dantzer R. (2001). Cytokine-induced sickness behavior: mechanisms and implications. *Ann. N. Y. Acad. Sci.* 933 222–234. 10.1111/j.1749-6632.2001.tb05827.x 12000023

[B12] Deak F. Sonntag W. E. (2012). Aging, synaptic dysfunction, and insulin-like growth factor (IGF)-1. *J. Gerontol. A Biol. Sci. Med. Sci.* 67 611–625. 10.1093/gerona/gls118 22503992 PMC3348499

[B13] Diz-Chaves Y. Astiz M. Bellini M. J. Garcia-Segura L. M. (2013). Prenatal stress increases the expression of proinflammatory cytokines and exacerbates the inflammatory response to LPS in the hippocampal formation of adult male mice. *Brain Behav. Immun.* 28 196–206. 10.1016/j.bbi.2012.11.013 23207108

[B14] Epstein I. Finkbeiner S. (2018). The Arc of cognition: Signaling cascades regulating Arc and implications for cognitive function and disease. *Semin. Cell Dev. Biol.* 77 63–72. 10.1016/j.semcdb.2017.09.023 29559111 PMC5865643

[B15] Fernández de Cossío L. Guzmán A. van der Veldt S. Luheshi G. N. (2017). Prenatal infection leads to ASD-like behavior and altered synaptic pruning in the mouse offspring. *Brain Behav. Immun.* 63 88–98. 10.1016/j.bbi.2016.09.028 27697456

[B16] Fletcher B. R. Hill G. S. Long J. M. Gallagher M. Shapiro M. L. Rapp P. R. (2014). A fine balance: Regulation of hippocampal Arc/Arg3.1 transcription, translation and degradation in a rat model of normal cognitive aging. *Neurobiol. Learn. Mem.* 115 58–67. 10.1016/j.nlm.2014.08.007 25151943 PMC4250373

[B17] Foster T. C. (2012). Dissecting the age-related decline on spatial learning and memory tasks in rodent models: N-methyl-D-aspartate receptors and voltage-dependent Ca2+ channels in senescent synaptic plasticity. *Prog. Neurobiol.* 96 283–303. 10.1016/j.pneurobio.2012.01.007 22307057 PMC3307831

[B18] Gautam A. Wadhwa R. Thakur M. K. (2016). Assessment of cholinergic properties of ashwagandha leaf-extract in the amnesic mouse brain. *Ann. Neurosci.* 23 68–75. 10.1159/000443573 27647956 PMC5020397

[B19] Guzowski J. F. Setlow B. Wagner E. K. McGaugh J. L. (2001). Experience-dependent gene expression in the rat hippocampus after spatial learning: a comparison of the immediate-early genes Arc, c-fos, and zif268. *J. Neurosci.* 21 5089–5098. 10.1523/jneurosci.21-14-05089.2001 11438584 PMC6762831

[B20] Hernandez A. R. Truckenbrod L. M. Campos K. T. Williams S. A. Burke S. N. (2020). Sex differences in age-related impairments vary across cognitive and physical assessments in rats. *Behav. Neurosci.* 134 69–81. 10.1037/bne0000352 31886694 PMC7078049

[B21] Huizink A. C. Robles de Medina P. G. Mulder E. J. Visser G. H. Buitelaar J. K. (2003). Stress during pregnancy is associated with developmental outcome in infancy. *J. Child Psychol. Psychiatry* 44 810–818. 10.1111/1469-7610.00166 12959490

[B22] Hussain S. Egbenya D. L. Lai Y. C. Dosa Z. J. Sørensen J. B. Anderson A. E. (2017). The calcium sensor synaptotagmin 1 is expressed and regulated in hippocampal postsynaptic spines. *Hippocampus* 27 1168–1177. 10.1002/hipo.22761 28686803

[B23] Inoue Y. Kamikubo Y. Ezure H. Ito J. Kato Y. Moriyama H. (2015). Presynaptic protein Synaptotagmin1 regulates the neuronal polarity and axon differentiation in cultured hippocampal neurons. *BMC Neurosci.* 16:92. 10.1186/s12868-015-0231-x 26667128 PMC4678605

[B24] Jakkamsetti V. Tsai N. P. Gross C. Molinaro G. Collins K. A. Nicoletti F. (2013). Experience-induced Arc/Arg3.1 primes CA1 pyramidal neurons for metabotropic glutamate receptor-dependent long-term synaptic depression. *Neuron* 80 72–79. 10.1016/j.neuron.2013.07.020 24094104 PMC3801421

[B25] Jin W. J. Feng S. W. Feng Z. Lu S. M. Qi T. Qian Y. N. (2014). Minocycline improves postoperative cognitive impairment in aged mice by inhibiting astrocytic activation. *Neuroreport* 25 1–6. 10.1097/WNR.0000000000000082 24247278

[B26] Kelly M. P. Deadwyler S. A. (2003). Experience-dependent regulation of the immediate-early gene arc differs across brain regions. *J. Neurosci.* 23 6443–6451. 10.1523/jneurosci.23-16-06443.2003 12878684 PMC6740623

[B27] Li X. W. Cao L. Wang F. Yang Q. G. Tong J. J. Li X. Y. (2016). Maternal inflammation linearly exacerbates offspring age-related changes of spatial learning and memory, and neurobiology until senectitude. *Behav. Brain Res.* 306 178–196. 10.1016/j.bbr.2016.03.011 26992827

[B28] Li X. Y. Wang F. Chen G. H. Li X. W. Yang Q. G. Cao L. (2016). Inflammatory insult during pregnancy accelerates age-related behavioral and neurobiochemical changes in CD-1 mice. *Age* 38:59 10.1007/s11357-016-9920-9923 PMC5005951 27194408

[B29] Li X. W. Chen G. H. Cao L. Yang Q. G. Wang F. (2018). Effects of early adulthood environmental factors intervention on learning and memory ability of the middle-aged CD-1 mice [CH]. *J. Anhui Agricult. Univ.* 2018 1028–1033.

[B30] Liu C. C. Wang J. Y. Tain Y. L. Chen Y. C. Chang K. A. Lai M. C. (2011). Prenatal stress in rat causes long-term spatial memory deficit and hippocampus MRI abnormality: differential effects of post-weaning enriched environment. *Neurochem. Int.* 58 434–441. 10.1016/j.neuint.2011.01.002 21215782

[B31] Lucassen P. J. Bosch O. J. Jousma E. Krömer S. A. Andrew R. Seckl J. R. (2009). Prenatal stress reduces postnatal neurogenesis in rats selectively bred for high, but not low, anxiety: possible key role of placental 11β-hydroxysteroid dehydrogenase type 2. *Eur. J. Neurosci.* 29 97–103. 10.1111/j.1460-9568.2008.06543.x 19032587

[B32] Marrone D. F. Satvat E. Shaner M. J. Worley P. F. Barnes C. A. (2012). Attenuated long-term Arc expression in the aged fascia dentata. *Neurobiol. Aging* 33 979–990. 10.1016/j.neurobiolaging.2010.07.022 20850902 PMC3010431

[B33] Maurer A. P. Johnson S. A. Hernandez A. R. Reasor J. Cossio D. M. Fertal K. E. (2017). Age-related changes in lateral entorhinal and CA3 neuron allocation predict poor performance on object discrimination. *Front. Syst. Neurosci.* 11:49. 10.3389/fnsys.2017.00049 28713251 PMC5491840

[B34] McAfoose J. Baune B. T. (2009). Evidence for a cytokine model of cognitive function. *Neurosci. Biobehav. Rev.* 33 355–366. 10.1016/j.neubiorev.2008.10.005 18996146

[B35] Newpher T. M. Harris S. Pringle J. Hamilton C. Soderling S. (2018). Regulation of spine structural plasticity by Arc/Arg3.1. *Semin. Cell Dev. Biol.* 77 25–32. 10.1016/j.semcdb.2017.09.022 28943393

[B36] Nolan A. M. Nolan Y. M. O’Keeffe G. W. (2011). IL-1β inhibits axonal growth of developing sympathetic neurons. *Mol. Cell. Neurosci.* 48 142–150. 10.1016/j.mcn.2011.07.003 21791245

[B37] Patterson S. L. (2015). Immune dysregulation and cognitive vulnerability in the aging brain: Interactions of microglia. IL-1β, BDNF and synaptic plasticity. *Neuropharmacology* 96 11–18. 10.1016/j.neuropharm.2014.12.020 25549562 PMC4475415

[B38] Plath N. Ohana O. Dammermann B. Errington M. L. Schmitz D. Gross C. (2006). Arc/Arg3.1 is essential for the consolidation of synaptic plasticity and memories. *Neuron* 52 437–444. 10.1016/j.neuron.2006.08.024 17088210

[B39] Ploski J. E. Pierre V. J. Smucny J. Park K. Monsey M. S. Overeem K. A. (2008). The activity-regulated cytoskeletal-associated protein (Arc/Arg3.1) is required for memory consolidation of Pavlovian fear conditioning in the lateral amygdala. *J Neurosci.* 28 12383–12395. 10.1523/JNEUROSCI.1662-08.2008 19020031 PMC6671728

[B40] Qiu J. Dunbar D. R. Noble J. Cairns C. Carter R. Kelly V. (2016). Decreased Npas4 and Arc mRNA levels in the hippocampus of aged memory-impaired wild-type but not memory preserved 11β-HSD1 deficient mice. *J. Neuroendocrinol.* 28. 10.1111/jne.12339 26563879 PMC4737280

[B41] Ramirez-Amaya V. Angulo-Perkins A. Chawla M. K. Barnes C. A. Rosi S. (2013). Sustained transcription of the immediate early gene Arc in the dentate gyrus after spatial exploration. *J. Neurosci.* 33 1631–1639. 10.1523/JNEUROSCI.2916-12.2013 23345235 PMC6618719

[B42] Sakaguchi Y. Sakurai Y. (2020). Left-right functional difference of the rat dorsal hippocampus for short-term memory and long-term memory. *Behav. Brain Res.* 382 112478. 10.1016/j.bbr.2020.112478 31935420

[B43] Schlotz W. Phillips D. I. (2009). Fetal origins of mental health: evidence and mechanisms. *Brain Behav. Immun.* 23 905–916. 10.1016/j.bbi.2009.02.001 19217937

[B44] Shipton O. A. El-Gaby M. Apergis-Schoute J. Deisseroth K. Bannerman D. M. Paulsen O. (2014). Left-right dissociation of hippocampal memory processes in mice. *Proc. Natl. Acad. Sci. U.S.A.* 111 15238–15243. 10.1073/pnas.1405648111 25246561 PMC4210314

[B45] Singh P. Thakur M. K. (2018). Histone Deacetylase 2 inhibition attenuates downregulation of hippocampal plasticity gene expression during aging. *Mol. Neurobiol.* 55 2432–2442. 10.1007/s12035-017-0490-x 28364391

[B46] Solas M. Aisa B. Mugueta M. C. Del Río J. Tordera R. M. Ramírez M. J. (2010). Interactions between Age. Stress and insulin on cognition: implications for alzheimer’s disease. *Neuropsychopharmacology* 35 1664–1673. 10.1038/npp.2010.13 20182419 PMC3055481

[B47] Thome J. Pesold B. Baader M. Hu M. Gewirtz J. C. Duman R. S. (2001). Stress differentially regulates synaptophysin and synaptotagmin expression in hippocampus. *Biol. Psychiatry* 50 809–812. 10.1016/s0006-3223(01)01229-x 11720700

[B48] Tong J. J. Chen G. H. Wang F. Li X. W. Cao L. Sui X. (2015). Chronic acarbose treatment alleviates age-related behavioral and biochemical changes in SAMP8 mice. *Behav. Brain Res.* 284 138–152. 10.1016/j.bbr.2015.01.052 25698601

[B49] Vanguilder H. D. Freeman W. M. (2011). The hippocampal neuroproteome with aging and cognitive decline: past progress and future directions. *Front. Aging Neurosci.* 23:8. 10.3389/fnagi.2011.00008 21647399 PMC3102218

[B50] Wadhwa P. D. Sandman C. A. Garite T. J. (2001). The neurobiology of stress in human pregnancy: implications for prematurity and development of the fetal central nervous system. *Prog. Brain Res.* 133 131–142. 10.1016/s0079-6123(01)33010-8 11589126

[B51] Wang F. Xu W. H. Wang C. Huang D. W. Chen G. H. (2010). Can outbred mice be used as a mouse model of mild cognitive impairment? *Neural Regen Res.* 5 1650–1656.

[B52] Wang H. L. Pei D. E. Yang R. D. Wan C. L. Ye Y. M. Peng S. S. (2019). Prenatal maternal vaginal inflammation increases anxiety and alters HPA axis signalling in adult male mice. *Int. J. Dev. Neurosci.* 75 27–35. 10.1016/j.ijdevneu.2019.04.001 30954504

[B53] Watanabe Y. Someya T. Nawa H. (2010). Cytokine hypothesis of schizophrenia pathogenesis: evidence from human studies and animal models. *Psychiatry Clin. Neurosci.* 64 217–230. 10.1111/j.1440-1819.2010.02094.x 20602722

[B54] Waung M. W. Pfeiffer B. E. Nosyreva E. D. Ronesi J. A. Huber K. M. (2008). Rapid translation of Arc/Arg3.1 selectively mediates mGluR-dependent LTD through persistent increases in AMPAR endocytosis rate. *Neuron* 59 84–97. 10.1016/j.neuron.2008.05.014 18614031 PMC2580055

[B55] Weinstock M. (2016). Prenatal stressors in rodents: Effects on behavior. *Neurobiol. Stress* 6 3–13. 10.1016/j.ynstr.2016.08.004 28229104 PMC5314420

[B56] Wu D. Bacaj T. Morishita W. Goswami D. Arendt K. L. Xu W. (2017). Postsynaptic synaptotagmins mediate AMPA receptor exocytosis during LTP. *Nature* 544 316–321. 10.1038/nature21720 28355182 PMC5734942

[B57] Yang J. Hou C. Ma N. Liu J. Zhang Y. Zhou J. (2007). Enriched environment treatment restores impaired hippocampal synaptic plasticity and cognitive deficits induced by prenatal chronic stress. *Neurobiol. Learn. Mem.* 87 257–263. 10.1016/j.nlm.2006.09.001 17049888

[B58] Zhao F. Liao Y. Tang H. Piao J. Wang G. Jin Y. (2017). Effects of developmental arsenite exposure on hippocampal synapses in mouse offspring. *Metallomics* 189 1394–1412. 10.1039/c7mt00053g 28901367

[B59] Zhu D. Li C. Swanson A. M. Villalba R. M. Guo J. Zhang Z. (2015). BAI1 regulates spatial learning and synaptic plasticity in the hippocampus. *J. Clin. Invest.* 125 1497–1508. 10.1172/JCI74603 25751059 PMC4396478

[B60] Zhu Z. Li X. Chen W. Zhao Y. Li H. Qing C. (2004). Prenatal stress causes gender-dependent neuronal loss and oxidative stress in rat hippocampus. *J. Neurosci. Res.* 78 837–844. 10.1002/jnr.20338 15499594

