# Supplementary material for: Effects of Prenatal Exposure to Inflammation Coupled With Stress Exposure During Adolescence on Cognition and Synaptic Protein Levels in Aged CD-1 Mice
Source: Front Aging Neurosci. 2020 Jul 6;12:157. doi: 10.3389/fnagi.2020.00157 (PMC7381390; doi:10.3389/fnagi.2020.00157)
Supplement: Supplementary file 1 [file Data_Sheet_1.docx]

Supplementary Material

# Supplementary Figures


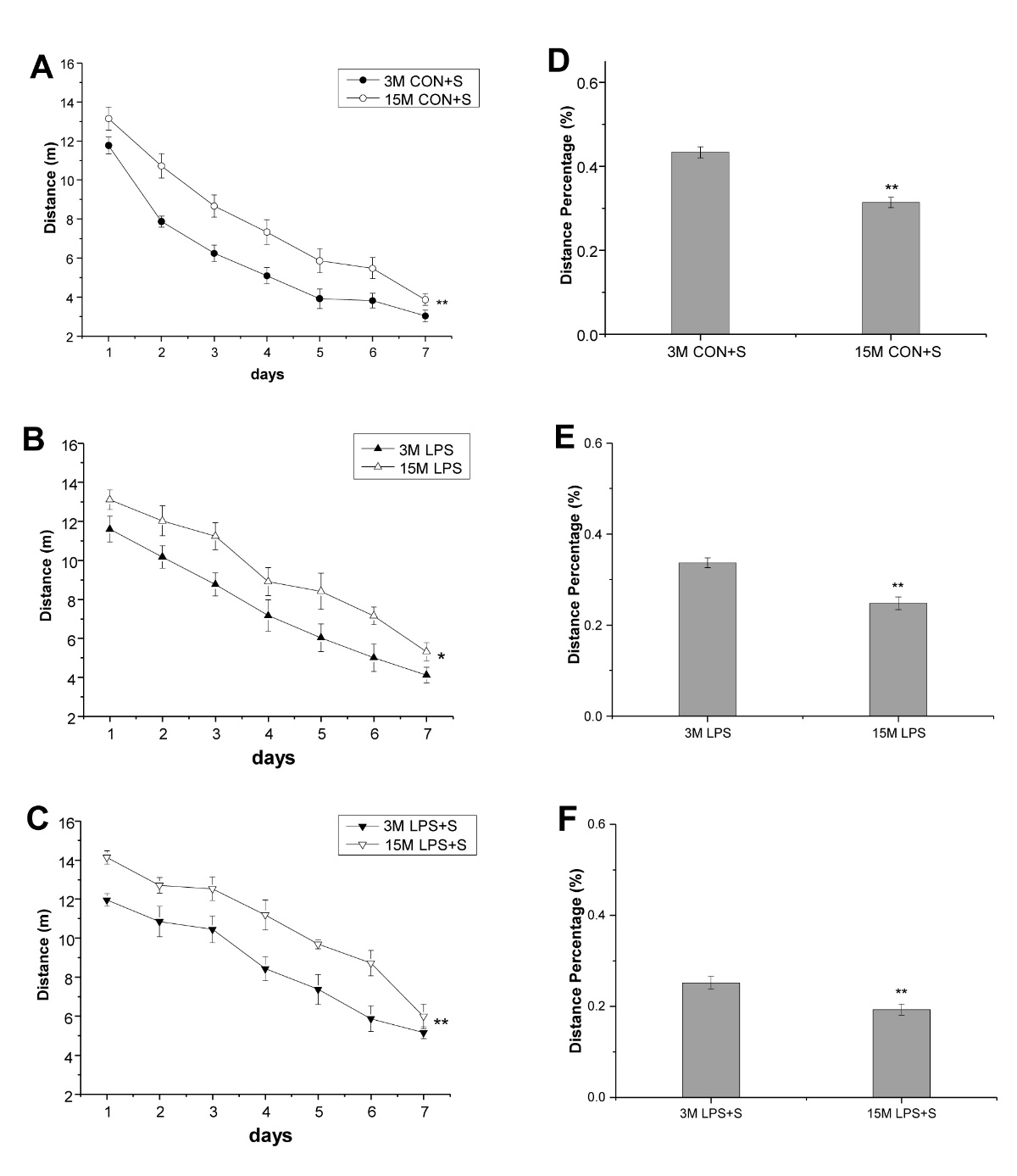


**Supplementary Figure 1.** The distance swam in the learning phase and the percent distance swam in the target quadrant in the memory phase of the MWM test in the different treatment groups, except untreated controls. The 15-month-old mice (15M) presented longer swimming distances and smaller distance percentages in the target quadrant than the 3-month-old mice (3M) in the CON+S (**A** and **D**), LPS (**B** and **E**), and LPS+S (**C** and **F**) groups. Error bars = SEM. Comparisons between groups in the same treatment, **P* < 0.05, ***P* < 0.01. MWM, Morris water maze; CON, untreated control; LPS, lipopolysaccharide treatment group; S, group of mice exposed to stress.


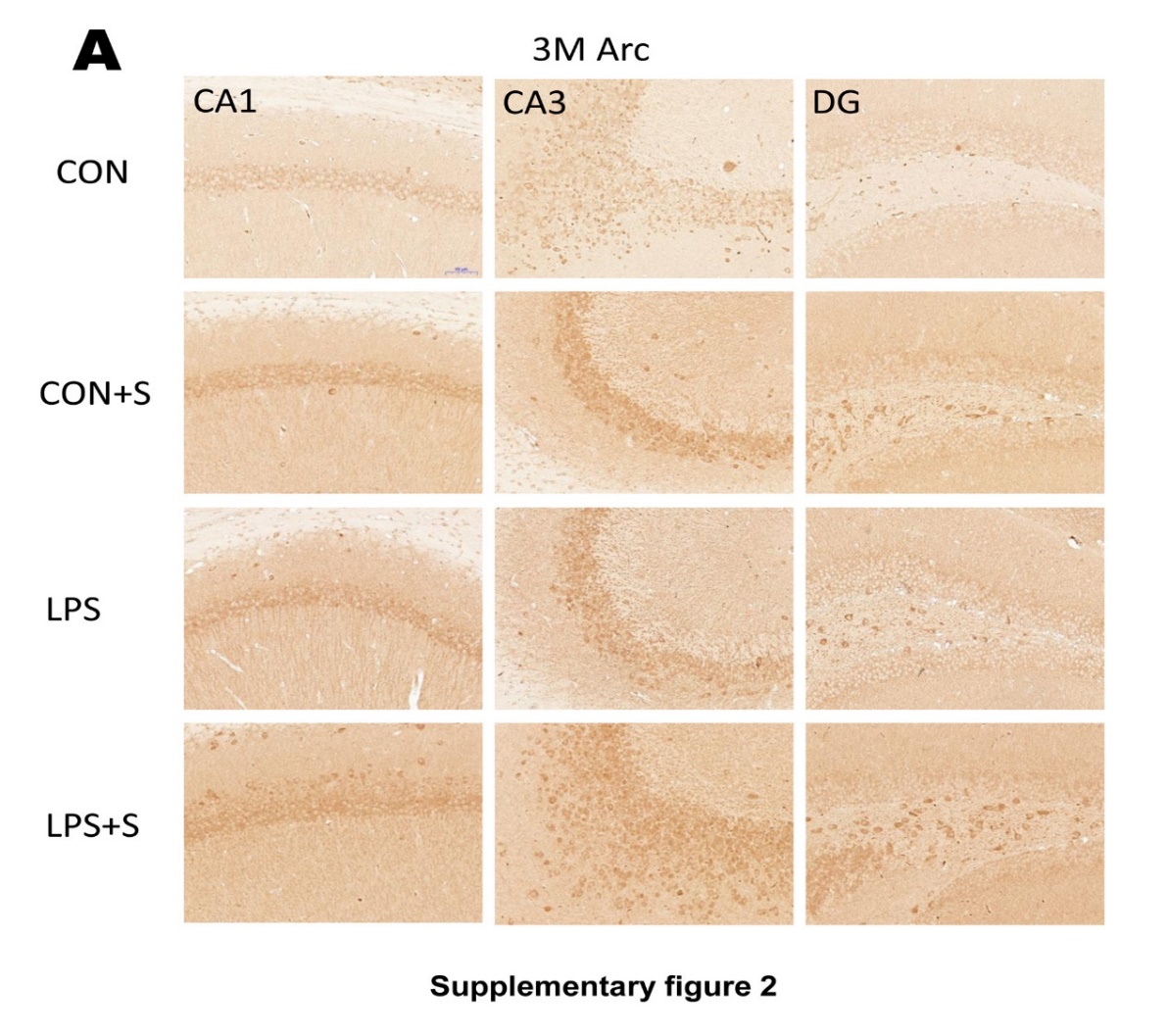

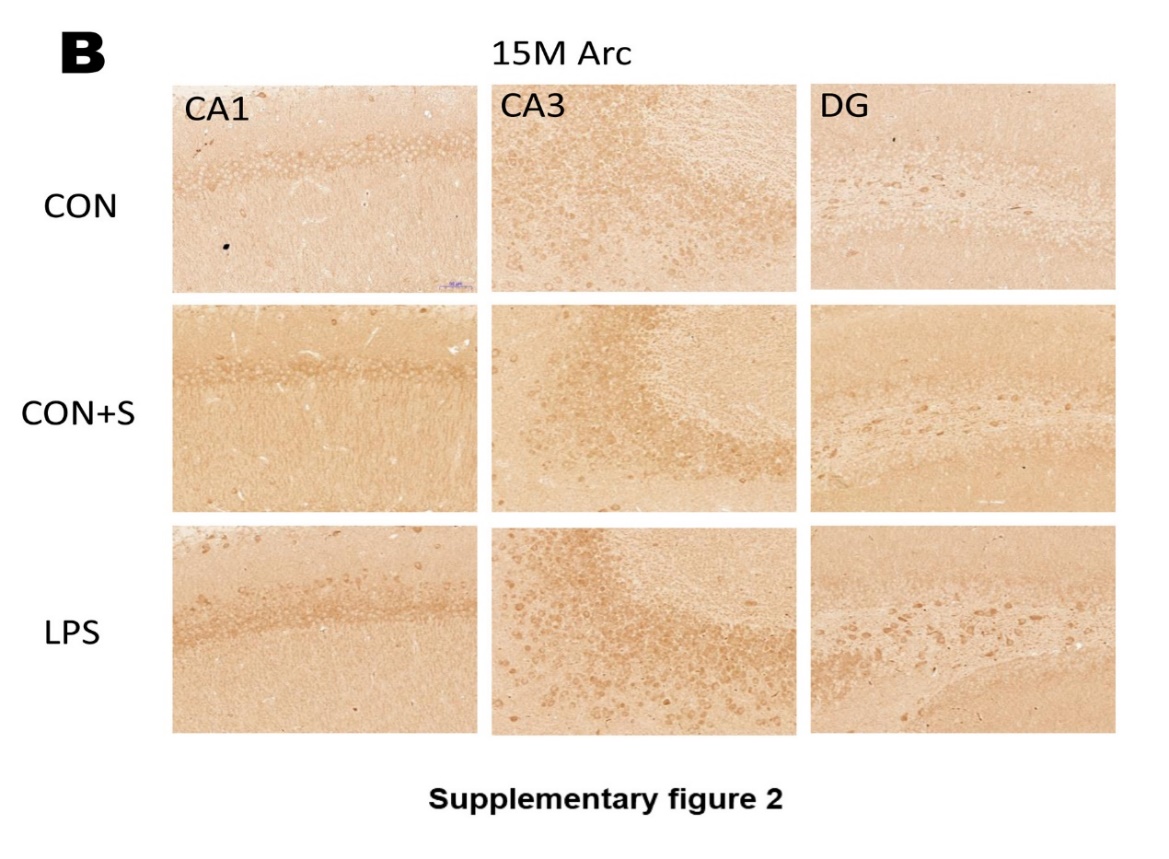


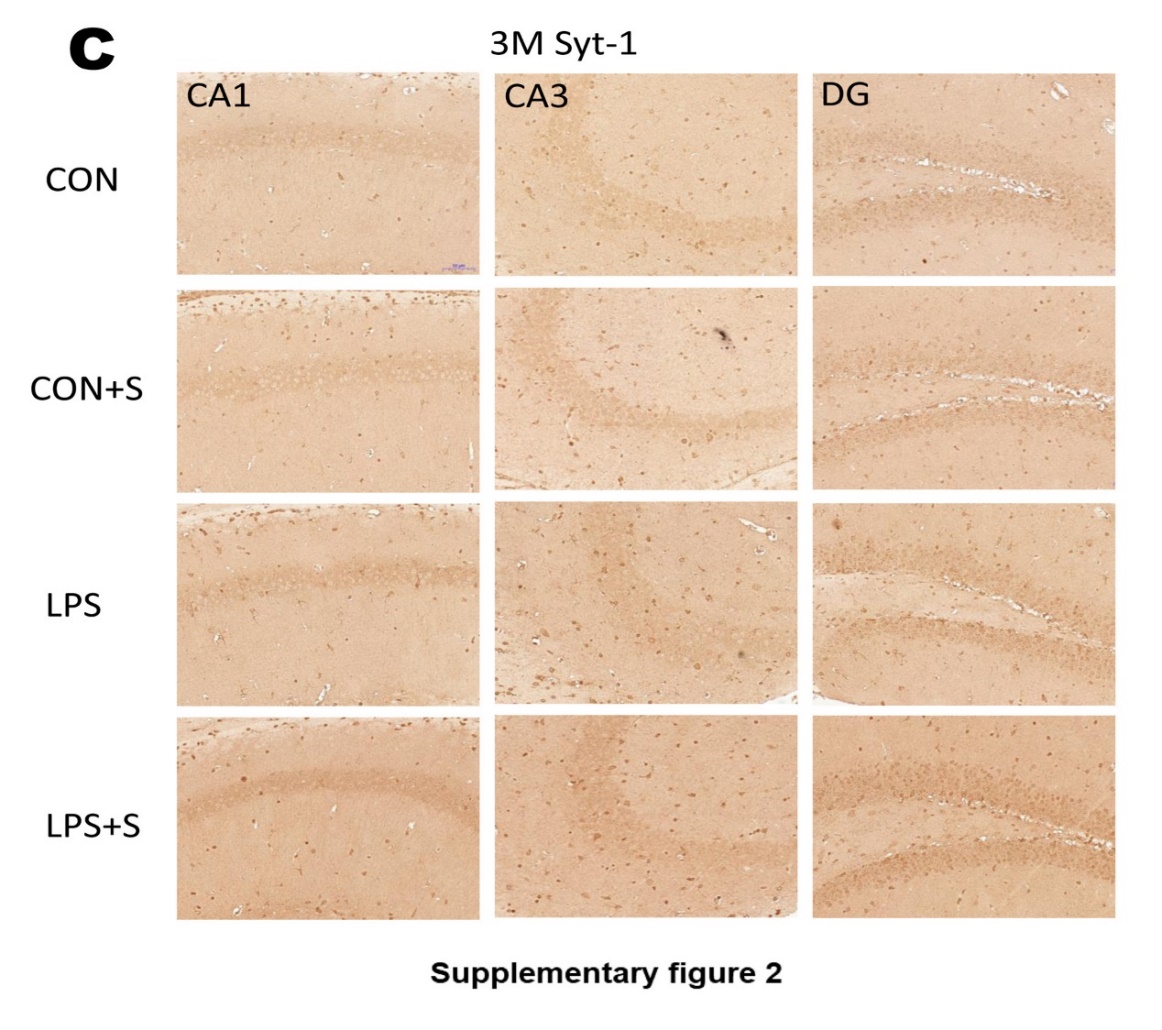


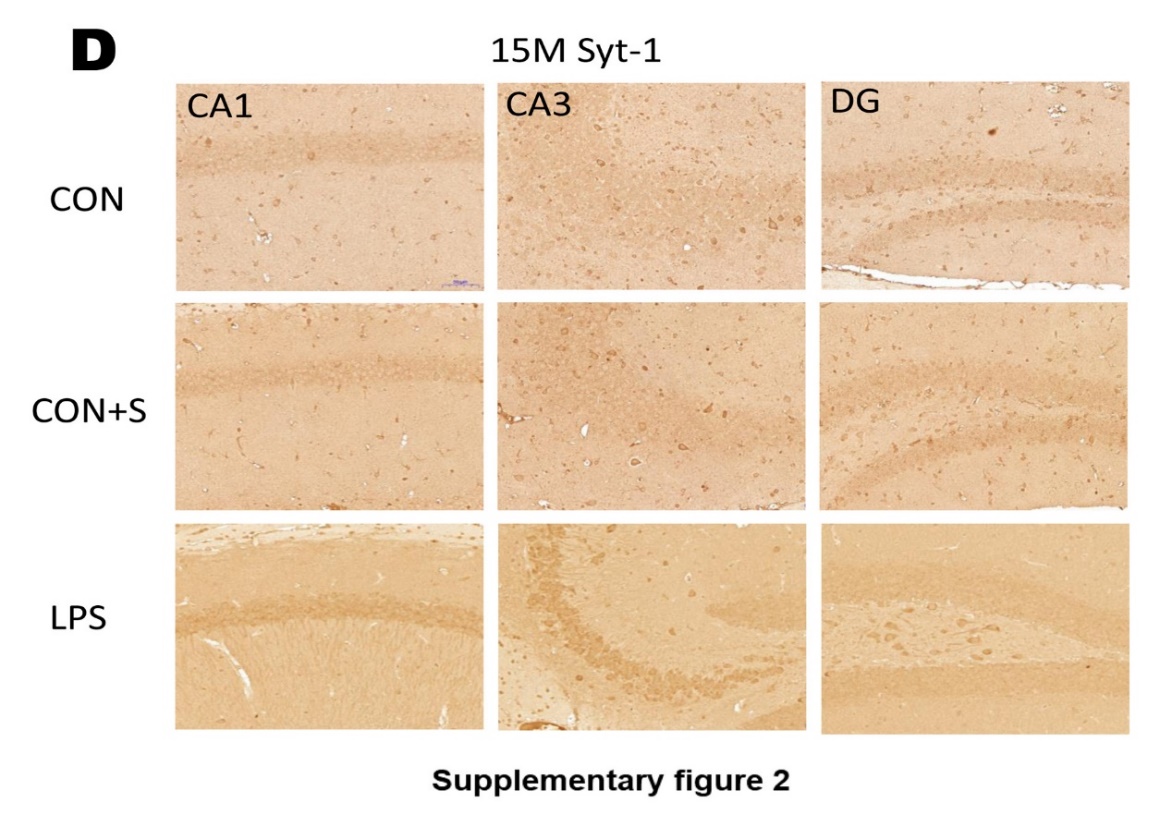


**Supplementary Figure 2.** Representative photomicrographs of Arc and Syt1 immunolabeling in the dorsal hippocampus and its subfields in 3- (3M) and 15-month-old (15M) CD-1 mice. (**A, B**) Arc immunoreactivity and (**C, D**) Syt1 immunoreactivity in the 3M (**A, C**) and 15M mice (**B, D**) from the different treatment groups.


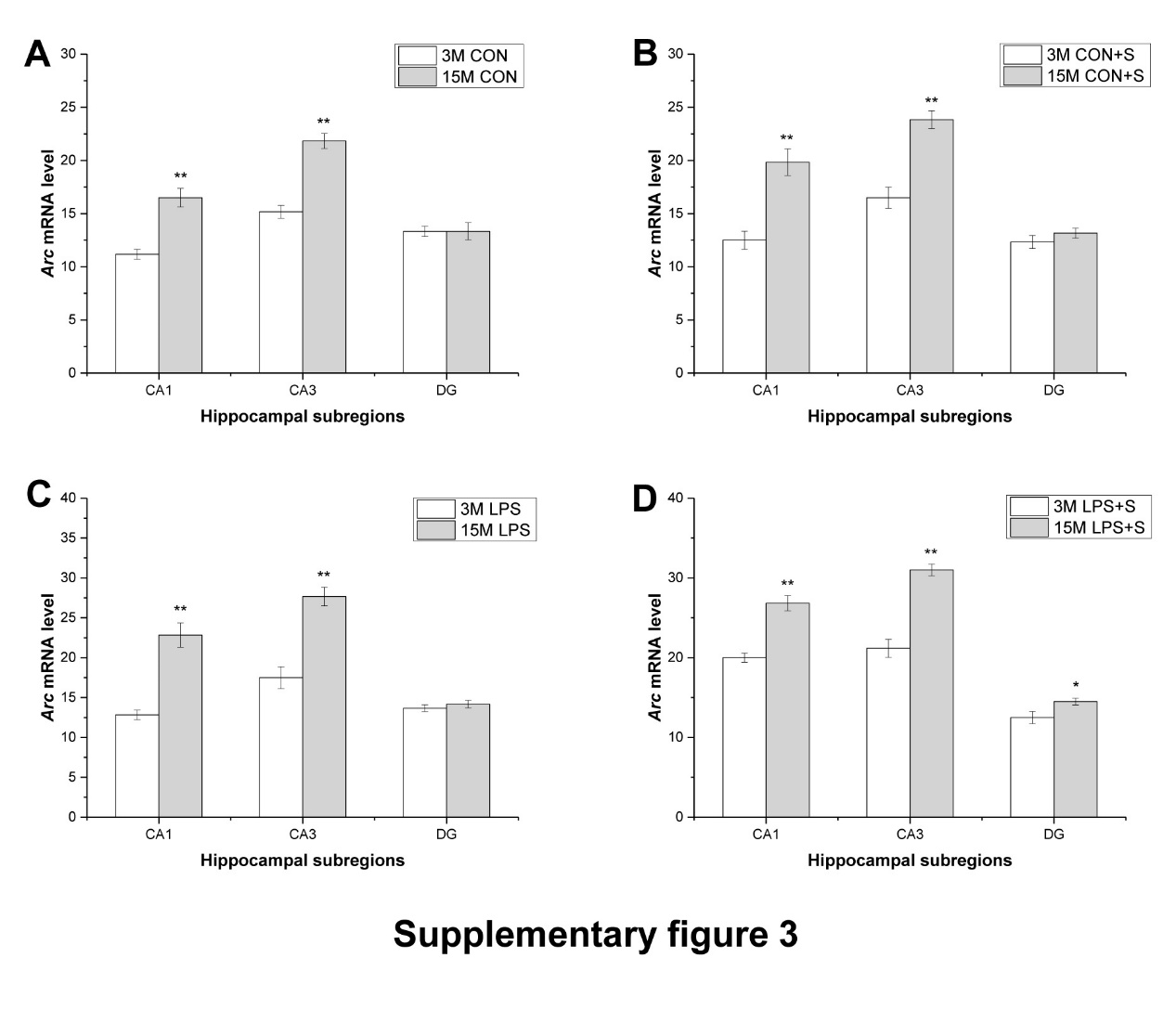


**Supplementary Figure 3.** The levels of *Arc* mRNA in different hippocampal subregions in the 3- (3M) and 15-month-old (15M) CD-1 mice. The 15M mice had higher *Arc* mRNA levels in the CA1 and CA3 subregions than the 3M mice in all the treatment groups [CON (**A**), CON+S (**B**), LPS (**C**), and LPS+S (**D**)]. Error bars = SEM. Comparison between groups in the same treatment, **P* < 0.05, ***P* < 0.01. CA, cornu ammonis; DG, dentate gyrus; CON, untreated control group; LPS, lipopolysaccharide treatment group; S, group of mice exposed to stress.


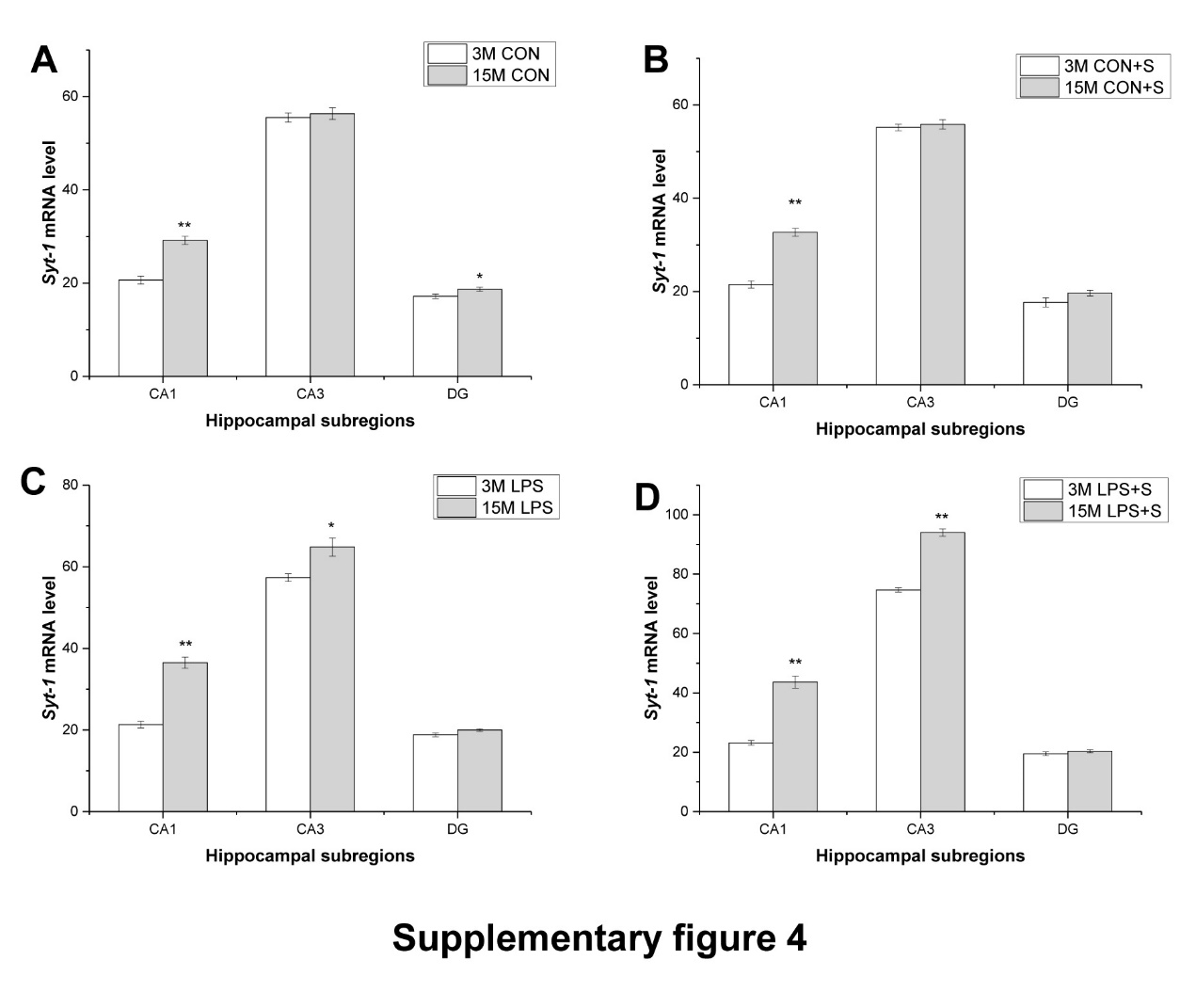


**Supplementary Figure 4.** The levels of *Syt1* mRNA in different hippocampal subregions in 3- (3M) and 15-month-old (15M) CD-1 mice. Compared with the 3M mice, the 15M mice had higher *Syt1* mRNA levels in the CA1 subregion in all the treatment groups, in the CA3 subregion in the LPS and LPS+S groups, and in the DG subregion in the CON group. Error bars = SEM. Comparison between groups in the same treatment, **P* < 0.05, ***P* < 0.01. CA, cornu ammonis; DG, dentate gyrus; CON, untreated control group; LPS, lipopolysaccharide treatment group; S, group of mice exposed to stress.


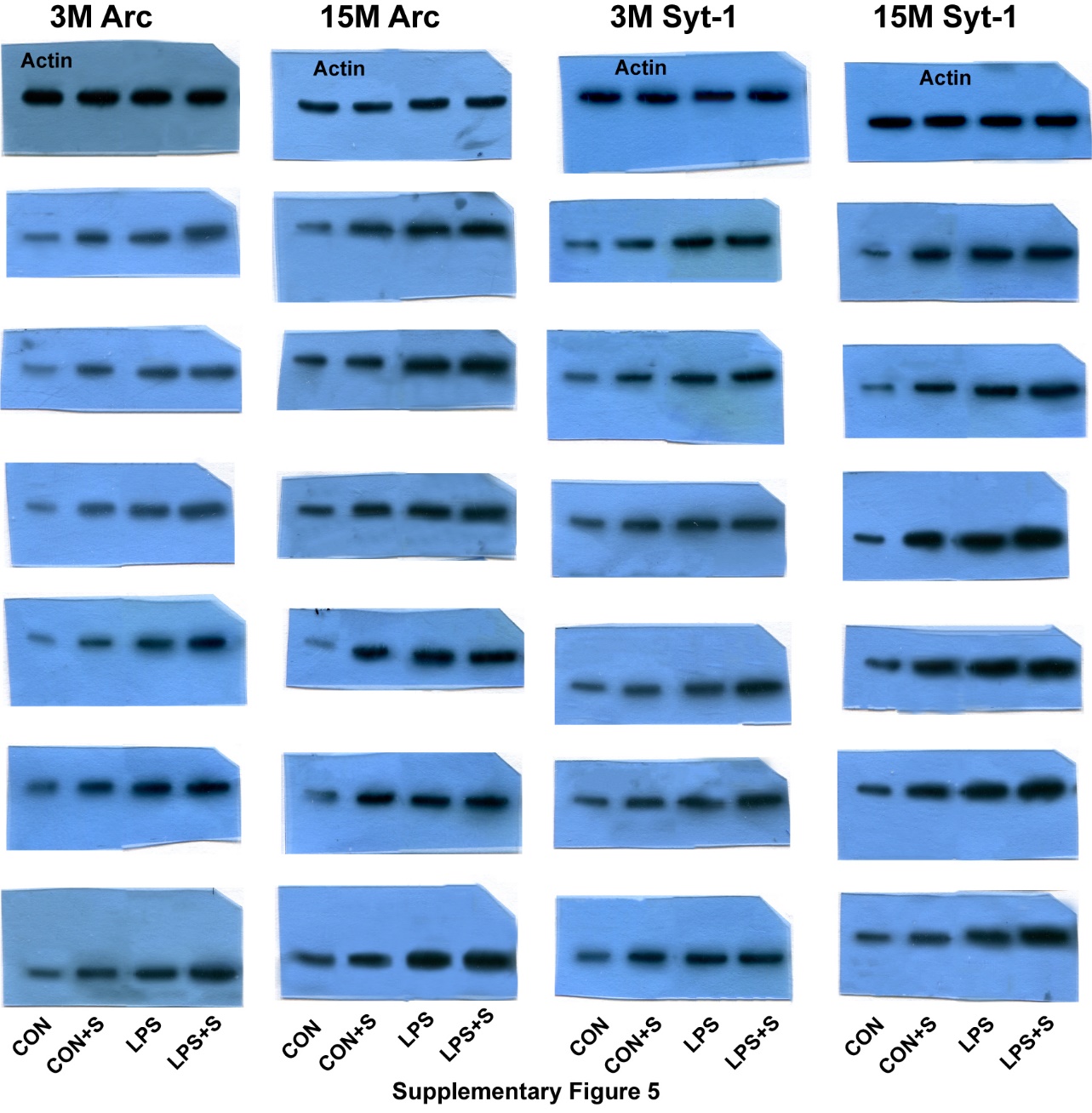


**Supplementary Figure 5.** The original images of Arc and Syt1 blots in 3- (3M) and 15-month-old (15M) CD-1 mice. Beta-actin was used as a control.
